# Unusual Secondary Metabolites from the Mangrove Ecosystems: Structures, Bioactivities, Chemical, and Bio-Syntheses

**DOI:** 10.3390/md20080535

**Published:** 2022-08-20

**Authors:** Meng-Jun Wu, Baofu Xu, Yue-Wei Guo

**Affiliations:** 1 State Key Laboratory of Drug Research, Shanghai Institute of Materia Medica, Chinese Academy of Sciences, 555 Zu Chong Zhi Road, Shanghai 201203, China; 2 Collaborative Innovation Center of Yangtze River Delta Region Green Pharmaceuticals and College of Pharmaceutical Science, Zhejiang University of Technology, Hangzhou 310014, China; 3 Shandong Laboratory of Yantai Drug Discovery, Bohai Rim Advanced Research Institute for Drug Discovery, Yantai 264117, China

**Keywords:** mangrove ecosystems, secondary metabolites, novel carbon skeletons

## Abstract

Mangrove ecosystems are widely distributed in the intertidal zone of tropical and subtropical estuaries or coasts, containing abundant biological communities, for example, mangrove plants and diverse groups of microorganisms, featuring various bioactive secondary metabolites. We surveyed the literature from 2010 to 2022, resulting in a collection of 134 secondary metabolites, and classified them into two major families in terms of the biological sources and 15 subfamilies according to the chemical structures. To highlight the structural diversity and bioactivities of the mangrove ecosystem-associated secondary metabolites, we presented the chemical structures, bioactivities, biosynthesis, and chemical syntheses.

## 1. Introduction

Identifying lead compounds is one of the biggest challenges in drug discovery. Natural products (NPs) and their intricate molecular frameworks have a long tradition as valuable starting points for drug development (for example, artemisinins, taxol, camptothecin, and penicillins). To date, NPs remain a significant source of new compounds, providing a wide range of structural diversities with multiple privileged scaffolds for drug discovery either directly, semi-synthetically, or as a source of inspiration [1,2,3,4,5]. However, discovering new bioactive NPs is generally time-consuming and laborious. Only a few new NP drug pharmacophores have been found over the past 20 years, representing critical issues for NPs-driven lead discovery campaigns.

Mangrove forests are complex ecosystems widely distributed in tropical and subtropical estuaries or coastal intertidal zones. These forests contain diverse biological communities, including mangrove plants and numerous groups of microorganisms. The environment of the mangrove system harbors unique traits, for instance, high salinity, low oxygen, tidal gradients, high temperature, and excessively intense light, resulting in an active ecosystem with various microorganisms [1,2,3,4,5]. Mangrove-associated microorganisms have been demonstrated to be a reliable source of bioactive metabolites and have likewise drawn the attention of NP researchers [6,7,8,9,10,11,12]. A large number of structurally unusual and bioactive NPs have been discovered from the mangrove-associated microorganisms, including fungal and bacterial endophytes isolated from the mangrove plants’ leaves, branches, and roots [13,14]. In addition, Mangrove sediments-derived microbes, a rich reservoir of NP diversity, could be utilized to explore new drugs [15].

The bioactive NPs solely from the true mangrove and semi-mangrove floras worldwide have been summarized in several review papers in 2010 [16,17]. However, the investigation of the mangrove ecosystem is mainly focused on the mangrove-associated microorganism but less on the mangrove flora in recent years. Three comprehensive reviews have recently focused on the NPs from mangrove-associated fungi and mangrove sediments-derived microbes [6,14,15]. However, to our knowledge, no review has been published on the secondary metabolites with unusual skeletons from the mangrove ecosystem. They might merit the attention of chemists and biologists and could be a source of fresh pharmacophores with biological activity for the creation of drugs based on natural products. 

In this review, we focus on the mangrove ecosystem-associated NPs featuring new carbon scaffolds, unique ring systems, or unusual functional moieties covering from 2010 to 2022. The structures, biological activities, biosynthesis, and total chemical syntheses of exampled unique compounds were included.

The references were searched using the following keywords as the subject search: natural products/secondary metabolites, mangrove, via Web of Science, Chemical Abstracts, and PubMed databases covering from 2010 to 2022, resulting in a collection of 134 unusual secondary metabolites. We classified them into two major families in terms of biological sources.

## 2. Unusual Natural Products from Mangrove Flora

### 2.1. Limonoids

Limonoids are natural tetranortriterpenoids containing a four-ring structure with a 17*β*-furyl ring mainly distributed in the Meliaceae and Rutaceae families [18]. In the mangrove flora, they are especially abundant and structurally diversified in the genus *Xylocarpus moluccensis* and *X. grantum* (family Meliaceae). Up to 2021, approximately 2700 limonoids have been identified. Moreover, owing to their widespread distribution and substantial content in Meliaceae plants, and active biosynthetic pathways, more than 1600, including 30 types of unique rearrangement skeletons, have been isolated and identified in the last 10 years [19]. Among them, nearly 233 new limonoids with 14 kinds of novel skeletons were isolated from mangrove *Xylocarpus*.

**Figure 1:**
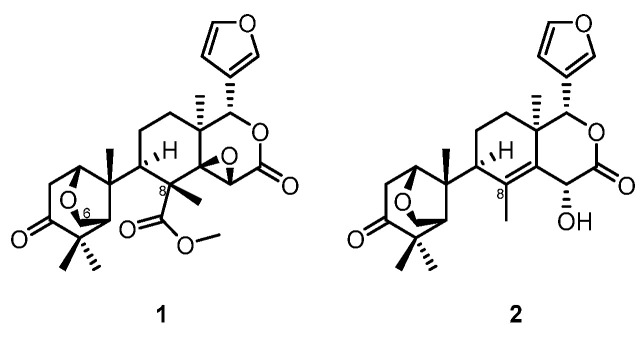
Structures of compounds **1** and **2**.

Thaixylomolin A (**1**), isolated from the seeds of a Thai mangrove *Xylocarpus moluccensis* collected at the Trang province, was obtained similar to the cleavage of C-6/C-7 by Baeyer–Villiger (BV) oxidation [20], and then the oxidized C-6 formed an unusual 6-oxabicyclo[3.2.1]octan-3-one motif with C-1 [21]. In 2016, the same research group isolated another analogue from *X. moluccensis*, thaixylomolin R (**2**) [22], whose C-8 is decarboxylated compared with **1** (Figure 1).

**Figure 2:**
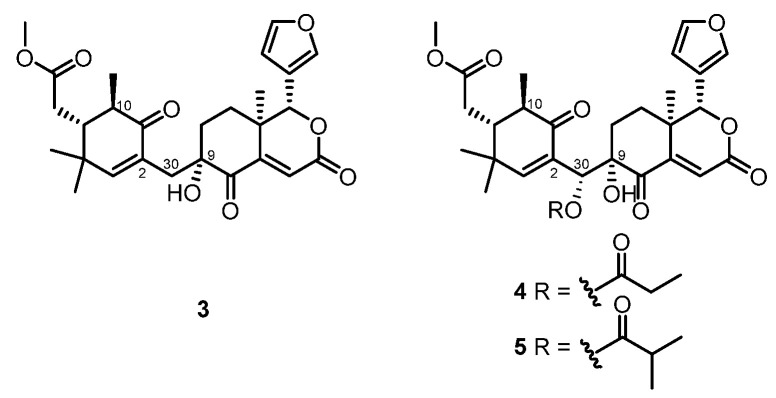
Structures of compounds **3**–**5**.

Xylomexicanin F (**3**) [23], hainangrantums I and J (**4** and **5**) [24] (Figure 2) are the second examples of a limonoid with an unusual 9, 10-*seco* and C-9-C-30 linkage, isolated from the seeds of the Chinese mangrove *X. granatum*. Among them, **3** showed moderate activity against the A549 and RERF cell lines with IC_50_ values of 18.83 *μ*M and 15.83 *μ*M, respectively. However, the first reported analogue, xylogranatin D, was concluded as an artifact [25,26].

**Scheme 1:**
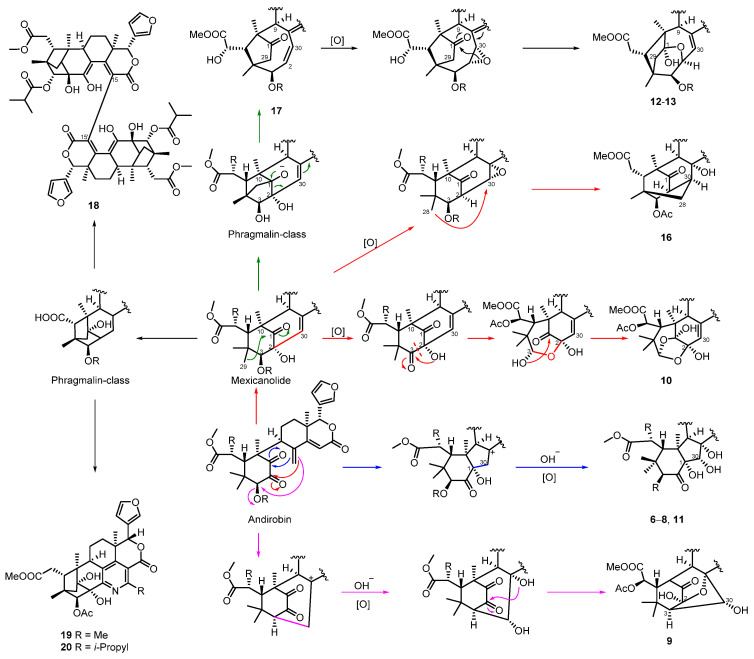
Proposed biosynthetic pathway for compounds **6**–**20** [21,27,29,30].

**Figure 3:**
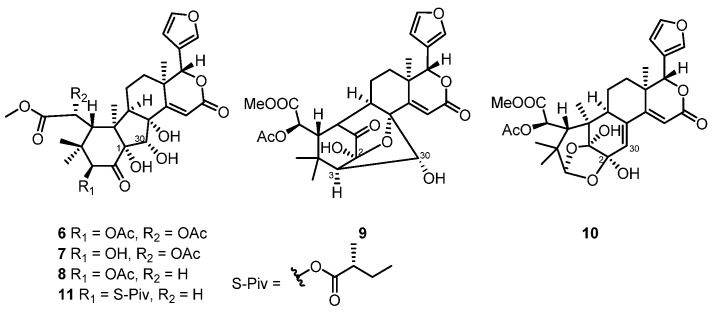
Structures of compounds **6**–**11**.

Chemical investigation of the seeds from a Trang (Thailand) mangrove *X. moluccensis* yielded five structurally intriguing limonoids, namely, trangmolins A–E (**6**–**10**) [27] (Figure 3). Notably, compounds **6**–**8** consist of unprecedented ring A/B-fused bicyclic moieties, and compound **10** represents the first example of the oxidative cleavage of the C2-C3 bond among limonoids. In 2021, a trangmolin A derivative krishnolide J (**11**) was isolated from seeds of the India Krishna mangrove *X. moluccensis* [28]. The biosynthetic origins of **6**–**11** could be traced back to a proposed andirobin-type limonoid with 1,2-bisketone groups [18]. Taking andirobin as the starting point, scientists from the Wu group proposed a biosynthetic pathway characterized by a highly divergent biosynthetic assembly line (Scheme 1) [27]. The three forks of the biosynthetic pathway obtain C-1/C-30 linkage (**6**–**8**, **11**), C-3/C-30 linkage (**9**), and C-2/C-30 linkage (**10**) based on the main mechanisms of electro- and nucleophilic enzymatic cascade reactions. The diverse cyclization patterns of **6**–**11** reveal the remarkable structural plasticity of rings A and B in limonoid biosynthesis.

**Figure 4:**
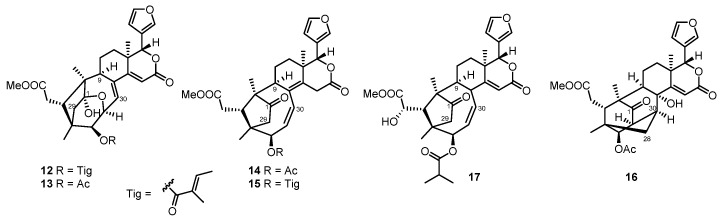
Structures of compounds **12**–**17**.

Andhraxylocarpins A–E (**12**–**16**) (Figure 4) were isolated and identified as three new types of limonoids from the seeds of two true mangroves, *X. granatum* (collected at the estuary of Krishna, India) and *X. moluccensis* (collected in the estuary of Godavari, India), respectively [29]. Among them, andhraxylocarpins A and B (**12** and **13**) contain an unprecedented 9-oxa-tricyclo-[3.3.2.1^7,10^]undecan-2-ene motif, andhraxylocarpins C-D (**14** and **15**) harbor a rare (Z)-bicyclo[5.2.1]dec-3-en-8-one substructure, and andhraxylocarpin E (**16**) possesses a unique tricyclo[3.3.1.1^3,6^]decan-9-one scaffold. In 2016, trangmolin F (**17**), which shared the same A/B fused carbobicyclic with **16**, was obtained from *X. moluccensis* by the same group [27] Wu et al. suggested a mexicanolide with a *Δ*^8,30^ double bond, derived from an andirobin by C-2/C-30 linkage and previously discovered among the genus *Xylocarpus*, may be the precursor of **12**–**16** [29] (Scheme 1). The presence of bridging rings (C10–C1–C2) in mexicanolide-type limonoids makes C-3 and C-30 close to each other in space, which leads to their coupling.

**Figure 5:**
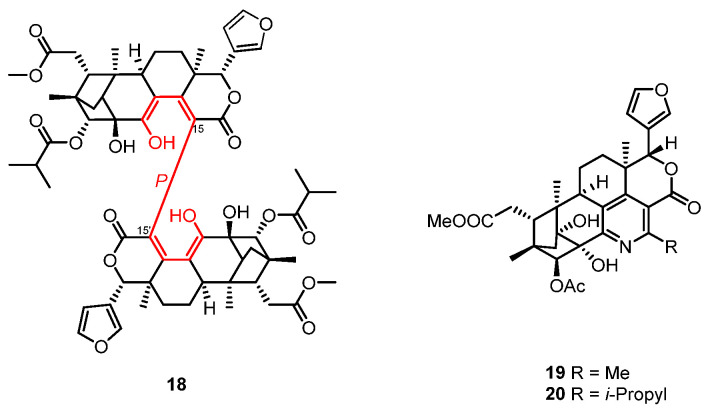
Structures of compounds **18**–**20**.

Krishnadimer A (**18**) (Figure 5) is the first dimeric limonoid isolated from the seeds of *X. moluccensis* with an unprecedented axial chirality architecture, with the C_2_-symmetric architecture, with a *P*-configured central axis at the C15, C15′-positions of the monomeric units, represents a milestone during decades of work on natural limonoids [30]. It could be obtained by the intermolecular oxidative coupling of the phargmalin-class limonoid, which can be derived from andirobin through C-2/C-30 and C-1/C-29 linkage. (Scheme 1) The semisynthesis of the dimer was successfully conducted. Subsequently, eight new limonoid dimers of four skeletons (two symmetric and two nonsymmetric) were designed and synthesized by oxidative carbon-carbon radical coupling [31].

Two unprecedented limonoids, thaixylomolins B and C (**19** and **20**) (Figure 5), co-isolates with **1** [21], are limonoids containing a unique pentasubstituted pyridine scaffold that might be generated by aromatization into a pyridine ring from a phargmalin-class limonoid. (Scheme 1) Thaixylomolin B (**19**) exhibited inhibitory activity against nitric oxide production in lipopolysaccharide and IFN-*γ*-induced RAW264.7 murine macrophages with an IC_50_ value of 84.3 *μ*M.

**Figure 6:**
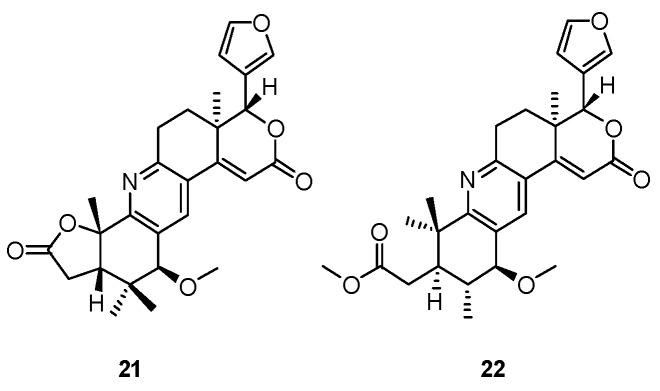
Structures of compounds **21** and **22**.

**Scheme 2:**
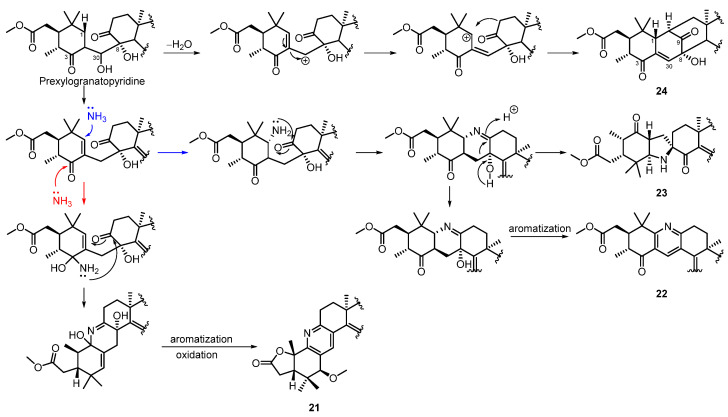
Proposed biosynthetic pathway for compounds **21**–**24 [23,32,33]**.

Two pyridine-containing limonoids, xylogranatopyridines A (**21**) and B (**22**) (Figure 6), were isolated from the twigs and leaves of *X. granatum*, collected from the seashore of Dongzhai, Hainan Province [32]. Compared to **21**, xylogranatopyridine B (**22**) possesses an unprecedented rearranged A-ring. Prexylogranatopyridine, a co-occurrence of limonoid with an unusual C-8-C-30 linkage, could be the common biosynthetic precursor of **21** and **22** (Scheme 2). Xylogranatopyridine A (**21**) exhibited significant inhibitory activity against protein tyrosine phosphatase 1B (PTP1B) with an IC_50_ value of 22.9 *μ*M.

**Figure 7:**
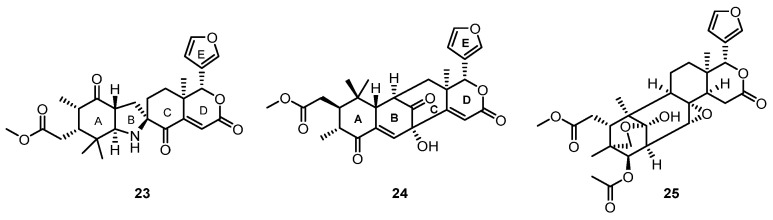
Structures of compounds **23**–**25**.

An unusual tetranortriterpenoid, xylomexicanins E (**23**) (Figure 7), which is the first example of limonoid with azaspiro skeleton between B (pyrrolidine) and C rings, was isolated from the seeds of the Chinese mangrove, *X. granatum* [23]. The plausible biosynthetic routes are proposed, as shown in Scheme 2, starting from the limonoid prexylogranatopyridine.

Further investigation of the seeds from the *X. granatum* led to the isolation of two new tetranortriterpenoids, xylomexicanins I and J (**24** and **25**) [33] (Figure 7). Notably, **24** represents an unprecedented limonoid with a bridged skeleton between the B- and C-rings, contrasting with analogues possessing bridged A- and B-rings (**25**). Wu et al. proposed that **24** was obtained from the same natural precursor as **22** after an enolate addition to the allylic alcohol moiety between C-3 and C-11 (Scheme 2).

**Figure 8:**
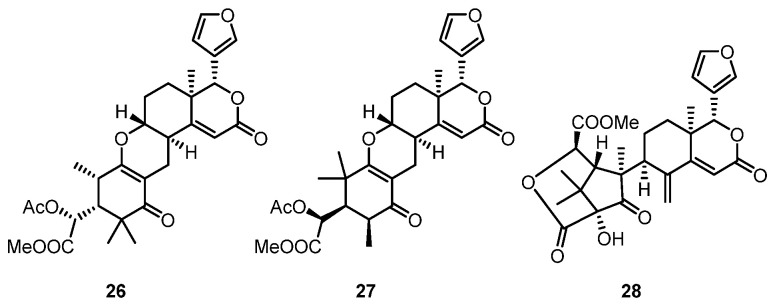
Structures of compounds **26**–**28**.

**Scheme 3:**
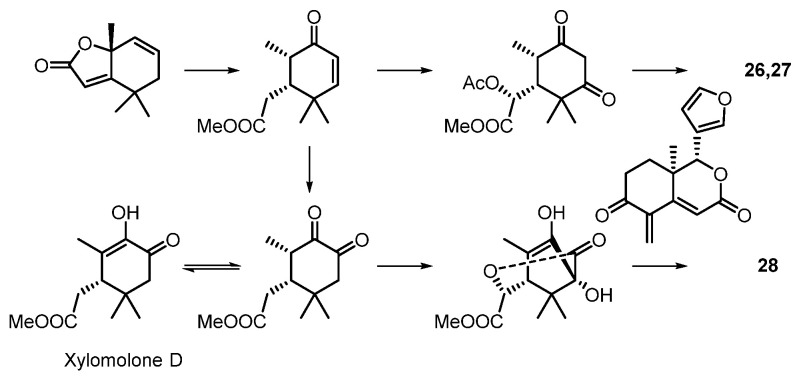
Proposed biosynthetic pathway for compounds **26**–**28** [34].

Three new limonoids, entitled xylomolones A–C (**26**–**28**, respectively, Figure 8) were discovered from the seeds of the Thai mangrove *X. moluccensis*, as well as a vital biosynthetic precursor, xylomolone D (a new C11-terpenic acid methyl ester) [34]. Compared to **26**, compound **27** is the first 9,10-*seco* limonoid with a 3,4-dihydro-2H-pyran motif and possesses the reversed alignment of ring A. For the biosynthesis of xylomolone C (**28**), a five-membered A-ring could be built through a benzylic acid-like rearrangement, forming an unusual 3-oxabicyclo[3.2.1]octan-2,7-dione motif; the C-2 is excluded from the A-ring in the rearrangement process. Wu et al. proposed a novel convergent strategy for limonoid biosynthesis (Scheme 3).

### 2.2. Diterpenoids

**Figure 9:**
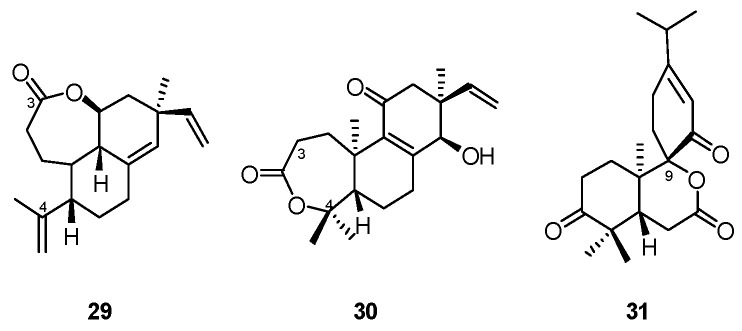
Structures of compounds **29**–**31**.

Two new *ent*-isopimarane-type diterpenoids agallochaexcoerins D and E (**29** and **30**) [35] (Figure 9), possessing an unusual seven-membered lactone moiety, were isolated from the wood of mangrove *Excoecaria agallocha*. Notably, **29** is the first report of *ent*-3,4-secoisopimaratriene diterpenoid with a rare seven-membered ring.

**Scheme 4:**
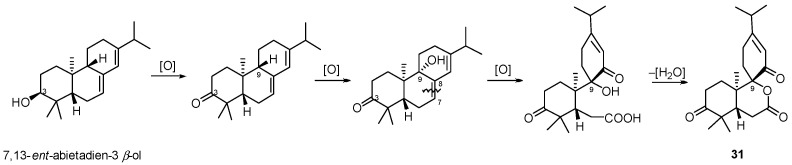
Proposed biosynthetic pathway for compound **31** [36].

Decandrinin (**31**) (Figure 9), an unprecedented C-9-spiro-fused 7,8-*seco*-*ent*-abietane, was obtained from the bark of an Indian mangrove *Ceriops decandra* (collected in the estuary of Godavari, Andhra Pradesh) [36]. The biosynthetic precursor might be the naturally more prevalent occurring 7,13-*ent*-abietadien-3*β*-ol and the plausible biosynthetic was proposed (Scheme 4). The spiro ring could be formed by oxidative cleavage and lactonization.

## 3. Unusual Natural Products from Mangrove-Associated Microorganisms

Previous chemical investigations of mangrove microbes especially mangrove-associated fungi resulted in the discovery of various bioactive secondary metabolites, including polyketides, terpenes, alkaloids, and peptides with diverse structural features.

### 3.1. Polyketides

Polyketides (PKs) are a large family of secondary metabolites with prominent structural diversity and various biological activities, isolated from diverse organisms. Polyketide synthases (PKSs) catalyze the sequential decarboxylative condensations of acyl-CoA thioesters to form structurally diverse PKs [37]. We direct readers to the fantastic reviews for more information on PKSs [38,39,40,41]. An even-increasing number of PKs from the mangrove-associated microorganisms are being reported.

#### 3.1.1. Coumarins and Isocoumarins

Naturally occurring coumarins and isocoumarins are an essential class of benzopyrene derivatives and are present in remarkable amounts in plants, while only a few are found in microorganisms and animal sources.

**Figure 10:**
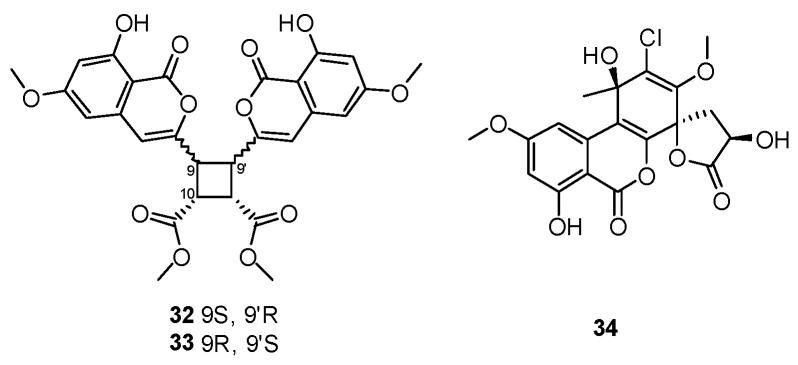
Structures of compounds **32**–**34**.

Up to now, 12 new coumarin and 102 new isocoumarin derivatives have been obtained from mangrove-associated fungi [42,43]. Among them, Peniisocoumarin A and B (**32** and **33**, Figure 10), a pair of unusual dimeric isocoumarin-type diastereoisomers containing a symmetric four-membered core at C-9/9′ and C-10/10′ were obtained from the fermentation of *Penicillium commune* QQF-3 (isolated from fresh fruit of the mangrove plant *Kandelia candel*) [44]. The structures of **32** and **33** were unanimously defined by X-ray diffraction analysis using Cu K*α* radiation. In 2015, Darsih et al., discovered penicilliumolide A (**34**) (Figure 10), an unusual tetracyclic isocoumarin containing a *γ*-lactone ring fused with a unique spiro framework, from the mangrove endophytic fungus *Penicillium chermesinum* HLit-ROR2 [45].

#### 3.1.2. Chromones

The chromone and its derivatives have been identified as the central backbone in several functional organic compounds, with strategic importance in many research and industrial domains. Until now, 85 new chromone derivatives have been identified from the mangrove-associated fungal species.

**Figure 11:**
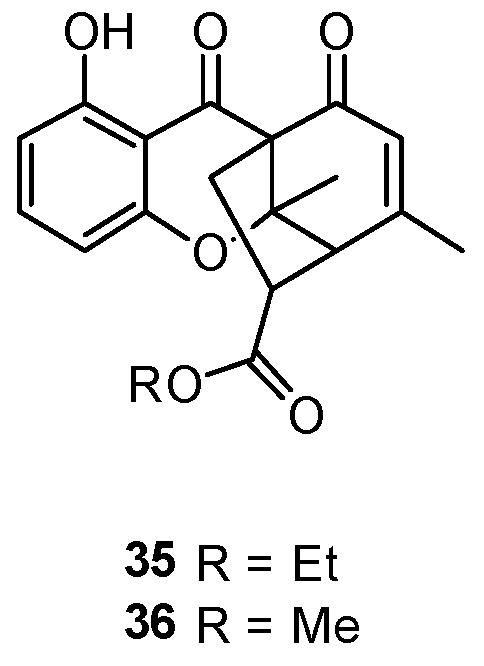
Structures of compounds **35** and **36**.

In 2019, two new polycyclic chromones, penixanthones C (**35**) and D (**36**) (Figure 11), possessing an unprecedented 6/6/6/5 polycyclic skeleton with a signature C_2_ bridge, were isolated from the mangrove sediment-derived fungus *Penicillium* sp. SCSIO041218 [46]. However, **35** and **36** only showed weak cytotoxicity. Furthermore, the configurations of **35** and **36** remain elusive.

#### 3.1.3. Azaphilones

Azaphilones or azaphilonoids are a structurally variable family of fungal polyketide metabolites harboring a highly oxygenated pyranoquinone bicyclic core [47]. In recent years, about 31 azaphilones with unusual structures and remarkable bioactivities were reported from mangrove-associated fungi, including genera *Aspergillus*, *Diaporthe*, *Penicillium*, and *Talaromyces*.

**Figure 12:**
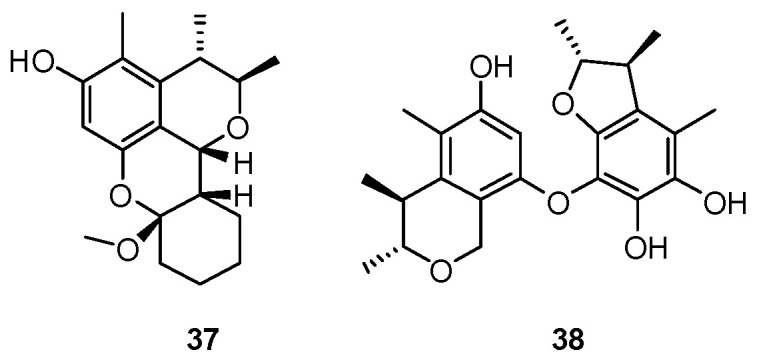
Structures of compounds **37** and **38**.

Two new citrinin derivatives, penicitol A (**37**) and penicitol B (**38**) (Figure 12) were identified from *Penicillium chrysogenum* ML226 obtained from the rhizosphere soil of the mangrove plant *Acanthus ilicifolius* [48]. The citrinin derivatives are a family of azaphilones, with the first one, namely citrinin, isolated from a *P. citrinum* strain in 1931 [49]. Penicitol A (**37**) is the first reported citrinin derivative with an unusual tetracyclic skeleton, and **38** is the first citrinin dimer with a single oxygen bridging center. **37** and **38** exhibited potent cytotoxic activities against HeLa, BEL-7402, HEK-293, HCT-116, and A-549 cell lines with IC_50_ values of 4.6–10.5 and 3.4–9.6 *μ*M, respectively. In 2011, Hosokawa et al. reported the first total synthesis of penicitol A (**37**), achieved by acetalization [50].

#### 3.1.4. Benzophenones Derivatives

Benzophenones (BPs) are widely distributed NPs possessing a diphenyl ketone moiety [51]. Given the presence of the chemically active carbonyl group, which can efficiently react with a variety of functional groups, diverse novel skeletons such as isobenzofuran, isoindolinone, and 3-dihydro-1H-indene, etc., could be formed. There are 12 members of BPs that are discovered from mangrove-associated fungi.

**Figure 13:**
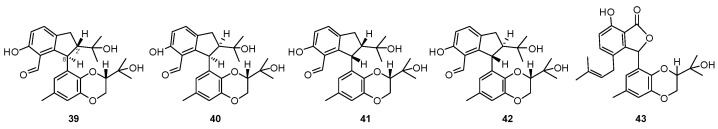
Structures of compounds **39**–**43**.

**Scheme 5:**
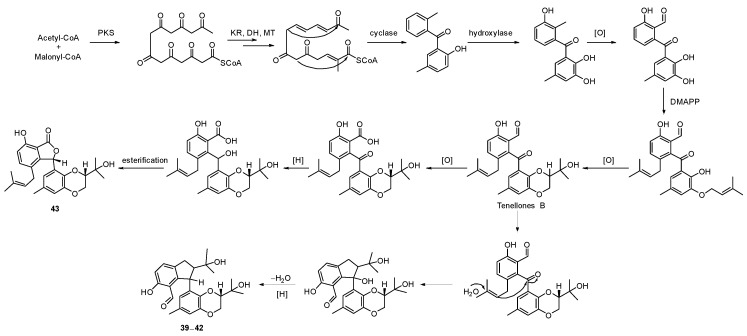
Proposed biosynthetic pathway for compounds **39**–**43** [53].

Four unusual 2,3-dihydro-1H-indene isomers, diaporindenes A–D (**39**–**42**) (Figure 13), and an unusual isoprenylisobenzofuran A (**43**) were isolated from *Diaporthe* sp. SYSU-HQ3, a fungus obtained from the branches of the mangrove plant *Excoecaria agallocha* collected from Zhuhai in Guangdong province, China [52]. Compounds **39**–**42** feature a 2,3-dihydro-1H-indene ring and a 1,4-benzodioxan moiety. Isoprenylisobenzofuran A (**43**) represented the first example of an isoprenylisobenzofuran nucleus possessing a rare 1,4-benzodioxan moiety. Biosynthetically, compounds **39**–**43** could be derived from co-occurrence benzophenone type metabolites such as tenellone B, which is formed by acetyl-CoA and malonyl-CoA through the catalysis of PKSs [53] (Scheme 5). In a bioassay, compounds **39**–**43** were found to exhibit significant inhibitory effects against nitric oxide production with IC_50_ values from 4.2–9.0 *μ*M and selective index (SI) values from 3.5 to 6.9.

#### 3.1.5. Macrolides

Macrolides, especially those possessing 10- to 19-membered ring systems, have diversified structural features and constitute a prominent group of active secondary metabolites. Since the discovery of well-known progenitor macrolide antibiotic pikromycin in 1950 and the second generation of macrolides such as azithromycin and clarithromycin, naturally occurring macrolides have been found today due to their diverse structures and promising biological properties [54]. A total of 63 macrolides have been isolated from the mangrove-associated fungi.

**Figure 14:**
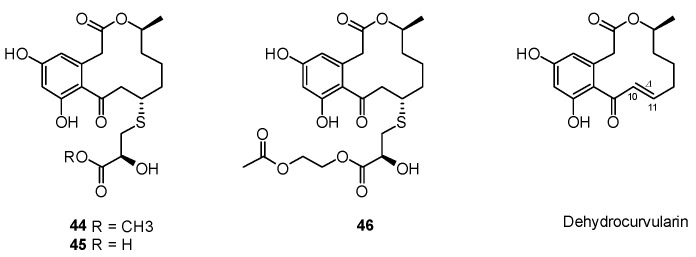
Structures of compounds **44**–**46** and dehydrocurvularin.

Sumalarins A−C (**44**−**46**) (Figure 14) were identified from the cytotoxic extract of *Penicillium. sumatrense* MA-92 from the rhizosphere of the mangrove *Lumnitzera racemose*. Notably, they were the unusual and rare examples of sulfur-containing curvularin derivatives isolated for the first time from natural sources [55]. Compounds **44**−**46** displayed cytotoxic activities against Du145, HeLa, Huh 7, MCF-7, NCI-H460, SGC-7901, and SW1990 cell lines with IC_50_ values ranging from 3.8 *μ*M to 10 *μ*M. Compound **44** is likely formed via Michael’s addition of 3-mercaptolactate to the double bond *Δ*^10,11^ of dehydrocurvularin. Esterification or acylation of **45** probably leads to the biosynthesis of **44** and **46** [55]. 

**Figure 15:**
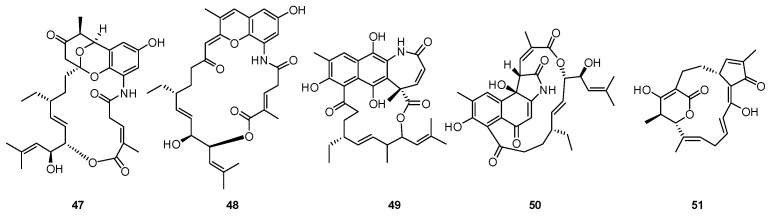
Structures of compounds **47**–**51**.

**Scheme 6:**
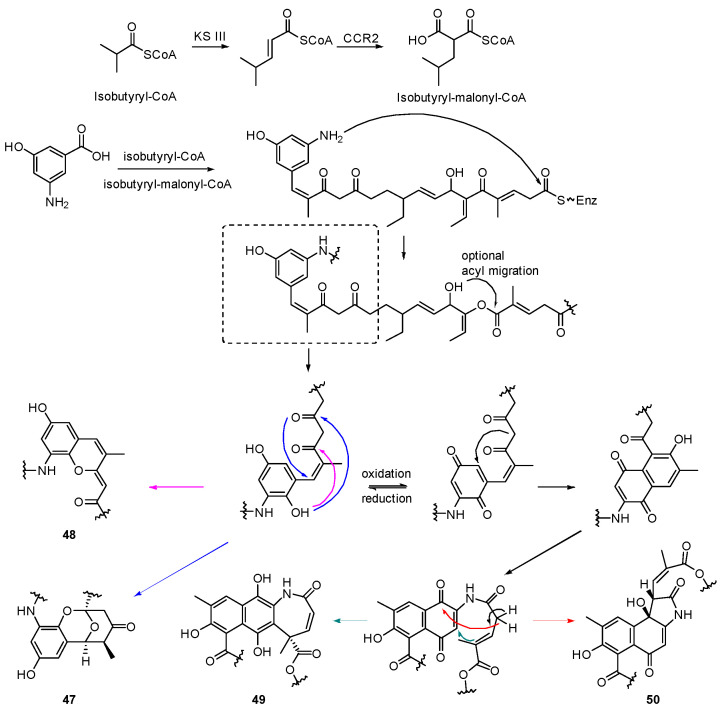
Proposed biosynthetic pathway for compounds **46**–**49** [56].

Ansamycins are characterized by an aromatic nucleus connected with a polyketide chain back to a nonadjacent position through an amide bond. Hertweck et al. isolated four unusual ansa macrolides, compounds **47**–**50** (Figure 15), from *Streptomyces* sp. HKI0576, a bacterial endophyte separated from the stem of mangrove *Bruguiera gymnorrhiza* [56]. This was the first report on discovering ansamycins from a plant endophyte. In addition, the degree of “in-built diversification” of these four compounds is unprecedented for complex polyketides. Among them, divergolide A (**47**) represents an unusual type of ansa macrolide with an unusual branched side chain and a disrupted polyketide backbone. Furthermore, the tricyclic chromophore is unprecedented for macrolides, and related *O*-heterocyclic substructures are only known from aromatic polyketides, such as the nogalamycin aglycone [57] and chaetoxanthone [58]. Divergolide B (**48**) represents another unusual type of ansa macrolide featuring a novel benzopyran/chromene core as the first congener of **47**. In addition, compounds **49** and **50** share substructures with **47** and **48** but feature structurally intriguing tetracyclic scaffolds. Furthermore, the ansa macrolides display significant antimicrobial and cytotoxic activities, probably regulating the immunity of the mangrove tree. Compounds **47**–**50** are biosynthesized from a common linear polyketide using 3-amino-5-hydroxybenzoic acid (AHBA) as a primer unit. Various reactions, including an optional acyl migration, generate the diverse multicyclic structures [56,59] (Scheme 6).

In 2014, Shen et al., cloned the biosynthetic gene cluster involved in the biosynthesis of the divergolides from the endophytic *Streptomyces* sp. W112 isolated from *Camptotheca acuminata*. Following gene disruption, gene overexpression, and bioinformatics analysis, they laid the foundation for further elucidation of the biosynthetic pathway as well as titer improvement [60]. In addition, Zhong et al. [61] conducted genome sequencing, bioinformatics analysis, and further isolations of four new divergolide congeners with a similar endophytic bacteria, *Streptomyces* sp. from *Bruguiera gymnorrhiza.* They showed that specialized acyltransferase domains are for selecting extender units, and the branched isobutylmalonyl-CoA is involved.

The total synthesis of divergolide A using the ring-closing metathesis (RCM) approach was published by Dai et al., in 2012 [62]. Subsequently, Rasapalli et al. synthesized the western section of divergolides C (**49**) and D (**50**) and demonstrated the robustness of C4-C5 as an appropriate approach for the further total synthesis of divergolides C and D in 2013 [63]. This chemical method was also conducted for divergolides A and B. Studies on the total synthesis of divergolides A-D using inexpensive, readily available starting materials and simple operations have also been constantly reported in recent years [64,65,66].

**Scheme 7:**
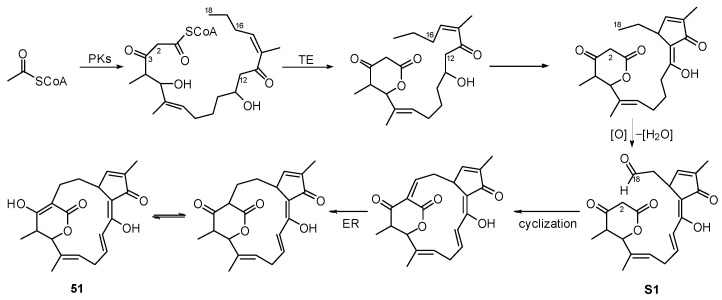
Proposed biosynthetic pathway for compound **51** [67].

A macrocyclic polyketide with an unusual carbon skeleton, namely hainanmycin A (**51**) (Figure 15), was isolated from *Streptomyces* sp. 219807 (from mangrove soil collected in Sanya) [67]. Compound **51** featured an unprecedented structural skeleton of a 17-membered carbocyclic framework. The cyclo-heptadeca framework containing a cyclopentenone ring substituted with a naturally occurring bridgehead enol motif is unique among NPs. It represents a new subgroup, a minor family of carbocyclic polyketide macrolides. Hong et al. [67] proposed a plausible biosynthetic pathway for **51** based on the biosynthesis of akaeolide [68], an analogue of **51**. Shortly, the PKS condenses acetyl-CoA and other building units (e.g., methylmalonyl-CoA and malonyl-CoA) to a linearized polyketide backbone. A thioesterase (TE) then releases the backbone with the formation of a *δ*-lactone ring. Further construction of the C-C bonds of C-16/C-12 and C-18/C-2 generate the structural core. Notably, a C-18 aldehyde intermediate (**S1**) might be involved in the C-18/C-12 carbon bond formation (Scheme 7).

#### 3.1.6. Others

**Figure 16:**
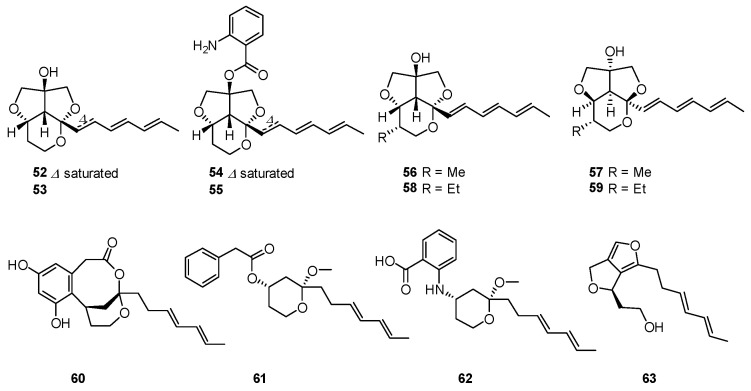
Structures of compounds **52**–**63**.

Eight new compounds, streptoglycerides A–H (**52**–**59**) (Figure 16) possessing a unique ring system, were obtained from *Streptomyces* sp. isolated from a mangrove sample collected on Kosrae Island [69,70]. This is the first report to describe a rare 6/5/5 tricyclic ring system consisting of a glycerol moiety from marine organisms. Streptoglyceride C (**54**) showed a weak inhibitory effect on nitric oxide production in BV-2 microglia cells. Compounds **56**–**59** showed significant anti-inflammatory activity by inhibiting lipopolysaccharide (LPS)-induced nitric oxide (NO) production in Raw 264.7 cells with IC_50_ values ranging from 3.5 to 10.9 *μ*M. It should be noted that **57** suppressed the transcription of iNOS and IL-6 without cytotoxicity.

**Scheme 8:**
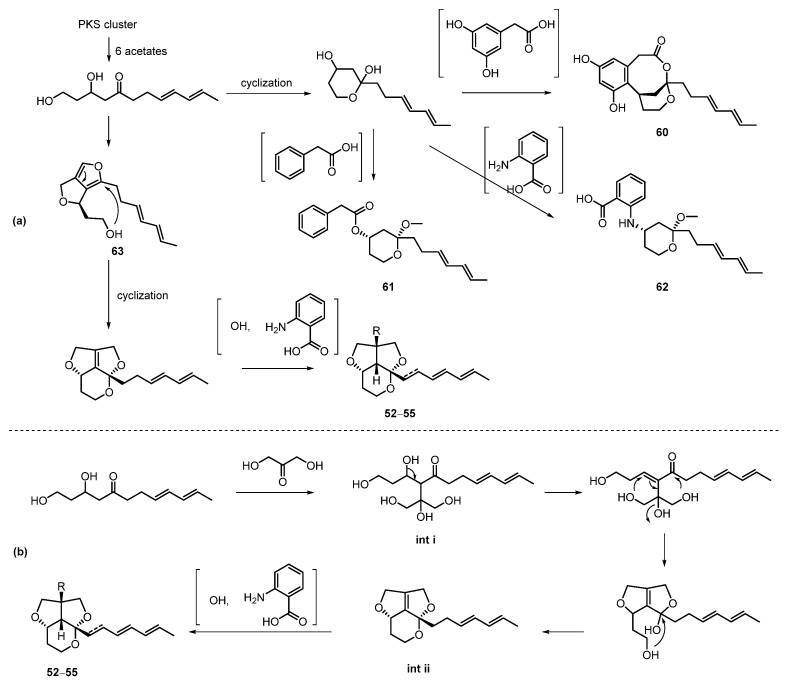
(**a**) Proposed biosynthetic pathway for compounds **52**–**55** and **60**–**63** in the literature [71]; (**b**) proposed biosynthetic pathway for **52**–**55** in this review.

Upon further investigation of the unusual strain, four new compounds, miharadienes A–D (**60**–**63**), possessing unique ring systems and a rare diene side chain, were isolated [71]. A plausible biosynthetic pathway of miharadienes and related compounds, streptoglycerides is proposed in the literature (Scheme 8a) [71]. However, the formation of **52**–**55** from **63** by attacking the nucleophilic hydroxy on the electron-rich furan ring seems inapplicable. Therefore, we proposed an optional pathway for **52**–**55** (Scheme 8b). In short, the starting lauryl alcohol derivative appears to react with dihydroxyacetone, an oxidation product of glycerol, to form the intermediate **int i** through Aldol type reaction of the C-4 active methylene of lauryl alcohol derivative with the carbonyl of the dihydroxyacetone. Then the hemiketal formation gives the tetrahydrofuran ring, and ether formation forms the other tetrahydrofuran ring. Afterward, the ketal formation by the interaction of the terminal hydroxyethylene with the hemiketal provides the pyran ring and affords the intermediate **int ii**, which could be further converted into **52**–**55**, possessing a rare 6/5/5 ring system.

### 3.2. Terpenoids

The new terpenoids from mangrove fungi can be divided into seven groups based on their chemical structures and biosynthetic pathways: monoterpenes, sesquiterpenes, diterpenes, sesterterpenes, triterpenes, and meroterpenes. Sesquiterpenes (138), sesterterpenes (36), and meroterpenes (72) comprise the most significant proportions of new terpenes from mangrove fungi. However, monoterpenes, diterpenes, and triterpenes were rarely isolated from mangrove fungi, and no new skeleton was discovered.

#### 3.2.1. Sesquiterpenoids

Sesquiterpenoids are the largest group of known terpenoids [72]. The mangrove fungi-derived sesquiterpenoids possess a variety of carbon skeletons, including monocyclic, bicyclic, and tricyclic types [73].

**Figure 17:**
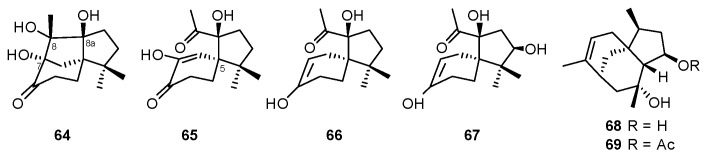
Structures of compounds **64**–**69**.

**Figure 18:**
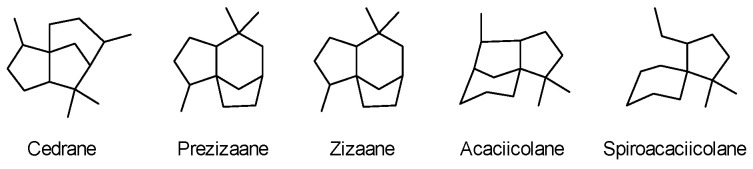
Structures of five sesquiterpene skeletons.

**Scheme 9:**
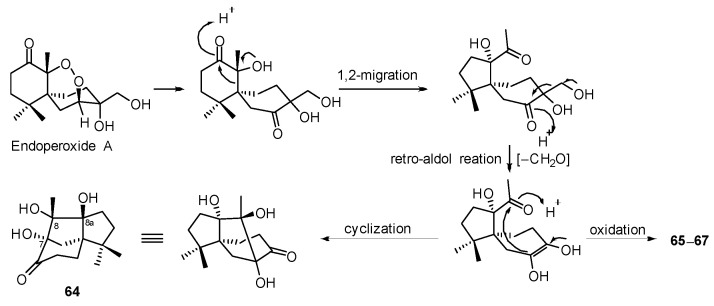
Proposed biosynthetic pathway for compounds **63**–**66** [74].

One tricyclic and three spirobicyclic norsesquiterpenoids (**64**–**67**) (Figure 17) were isolated from the endophytic fungus *Pseudolagarobasidium acaciicola* (from the mangrove *Bruguiera gymnorrhiza*) [74,75]. Among them, acaciicolin A (**64**) possesses a previously unknown skeleton with a uniquely connected 6/5/5 ring system and three consecutive oxygenated sp^3^ quaternary carbons at C-7, C-8, and C-8a. The norsesquiterpene skeleton of **64** was named “acaciicolane”, and was different from the three known sesquiterpene skeletons with 6/5/5 ring systems: cedrane, prezizaane, and zizaane (Figure 18). Spiroacaciicolides A–C (**65**–**67**) has a hitherto unobserved 5/6 fused spirobicyclic ring system. The absolute configurations of the new compounds **64**–**66** were determined by single-crystal X-ray analysis (Cu-K*α* radiation). **64**–**67** could originate from chamigrane endoperoxide A [76] (Scheme 9).

**Scheme 10:**
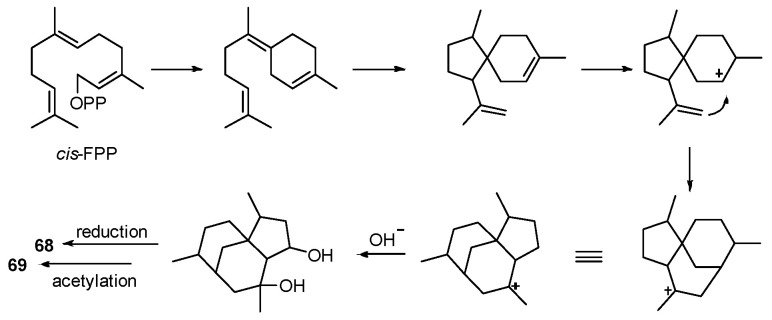
Proposed biosynthetic pathway for compounds **68** and **69** [77].

Penicibilaenes A (**68**) and B (**69**) (Figure 17), two sesquiterpenes possessing a tricyclo[6.3.1.0^1,5^]dodecane skeleton constituted by [3.3.1]-bridged and [4.3.0]-fused junctions, were characterized from *Penicillium bilaiae* MA-267, a fungus obtained from the rhizospheric soil of the mangrove plant *Lumnitzera racemosa* [77]. An X-ray crystallographic study determined the structure and configuration. The hypothetical biosynthetic pathway starting from *cis*-farnesyl pyrophosphate (FPP) was proposed (Scheme 10). Notably, Compounds **68** and **69** exhibited selective activity against the plant pathogenic fungus *Colletotrichum gloeosporioides* (MIC = 1.0 and 0.125 *μ*g/mL, respectively).

The first total synthesis of **68** and **69** in their racemic forms was reported by Dong et al. in 2021 [78]. The approach featured a rhodium-catalyzed deconstructive formation of a tricyclic skeleton by C–C activation of cyclobutanone derivatives, generating (±)-**68** and (±)-**69** in 13 and 14 steps with 0.56% and 0.49% overall yields, respectively. In the same year, K Sugita described another more efficient synthetic pathway for the total practical synthesis of (±)-**68** and (±)-**69** from commercially available 3-ethoxycyclohex-2-en-1-one with 4.0% overall yields for both compounds [79].

#### 3.2.2. Sesterterpenoids

Sesterterpenoids are a relatively small and rare group of terpenoids found in widespread sources. They always possess interesting carbon skeletons, including linear, monocyclic, polycyclic, and miscellaneous. In addition, they exhibit diverse biological activities such as antimicrobial, cytotoxicity, anti-inflammatory, and protein tyrosine phosphatase B inhibition.

**Figure 19:**
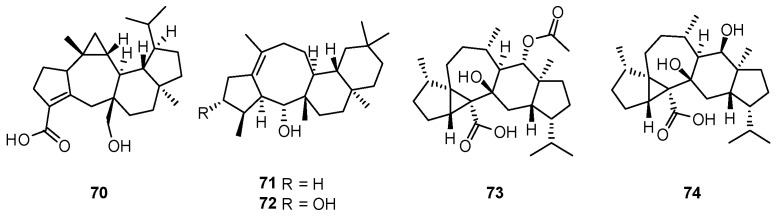
Structures of compounds **70**–**74**.

The group of She has been dedicated to the search for structurally unique and biologically active compounds from the mangrove plant-derived fungal endophytes. Five sesterterpenoids of three kinds of carbon skeletons, asperterpenoid A (**70**) (Figure 19), asperterpenols A and B (**71** and **72**), and aspterpenacids A and B (**73** and **74**), have been isolated and characterized from *Aspergillus* sp. Among them, asperterpenoid A (**70**), possessing a new 5/7/(3)6/5 pentacyclic carbon skeleton, exhibited potent inhibitory activity against *Mycobacterium tuberculosis* protein tyrosine phosphatase B (mPTPB) with an IC_50_ value of 2.2 *μ*M [80].

**Scheme 11:**
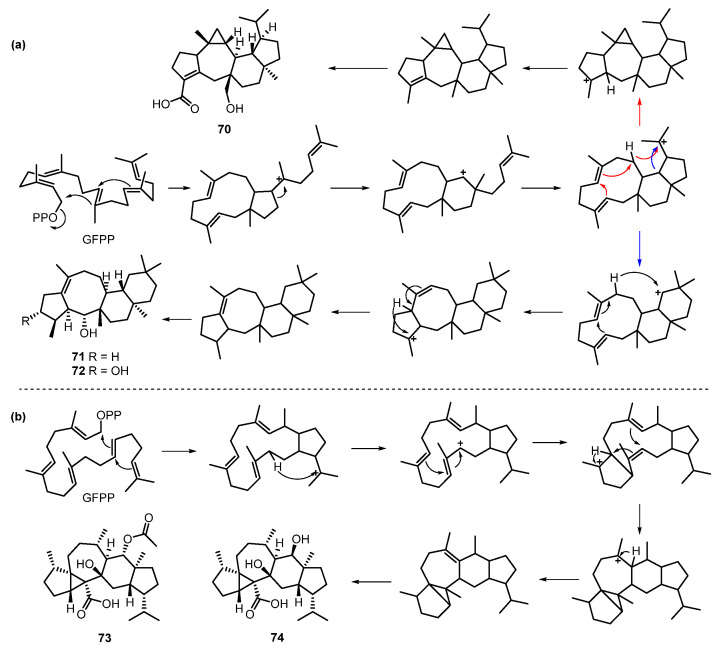
(**a**) Proposed biosynthetic pathway for compounds **70**–**72** [80,81]; (**b**) Proposed biosynthetic pathway for compounds **73**–**74** [82].

In addition, asperterpenol A (**71**) and asperterpenol B (**72**), two sesterterpenoids with an unusual 5/8/6/6 tetracyclic ring skeleton, showed inhibitory effects on acetylcholinesterase (AChE) with IC_50_ values of 2.3 *μ*M and 3.0 *μ*M, respectively [81]. Furthermore, the two unusual pentacarbocyclic sesterterpenoids, aspterpenacids A (**73**) and B (**74**), with an unusual carbocyclic skeleton containing an unprecedented 5/3/7/6/5 ring system, showed no antibacterial and cytotoxic activities [82]. The structures of **69**–**73** were elucidated based on spectroscopic methods, and the absolute configurations of **70**–**73** were determined by single-crystal X-ray diffraction analysis. She et al. proposed a hypothetical biosynthetic pathway for **70**–**74** [80,81,82]. In brief, they are derived from geranylfarnesyl pyrophosphate (GFPP), followed by cyclization rearrangement and redox reactions (Scheme 11).

#### 3.2.3. Meroterpenoids

Meroterpenoids are secondary metabolites with structures consisting of at least two parts: a terpenoid fragment (mainly mevalonate pathway) and a nonterpenoid fragment [83]. The different nonterpenoid moiety based on the biosynthetic pathway, various terpenoid (the length of the terpenoid chain and its cyclization mode), and the tailoring reactions make the chemical diversity of meroterpenoids.

**Figure 20:**
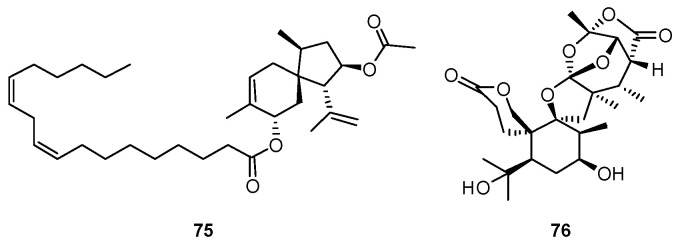
Structures of compounds **75** and **76**.

Chermebilaene A (**75**) (Figure 20), an unprecedented acorane-type sesquiterpene hybridized with an octadecadienoic acid skeleton, together with an unusual orthoester meroterpenoid, chermebilaene B (**76**) were isolated from the co-culture extract of *P. bilaiae* MA-267 (from the rhizosphere of the mangrove *Lumnitzera racemosa*) and *P. chermesinum* EN-480 (from the fresh tissue of marine red algal *Pterocladiella tenuis*) [84]. Compound **75** showed potent inhibitory activities against *Ceratobasidium cornigerum* and *Edwardsiella tarda*, and may prove helpful as an antibiotic against aquatic or plant pathogens.

**Figure 21:**
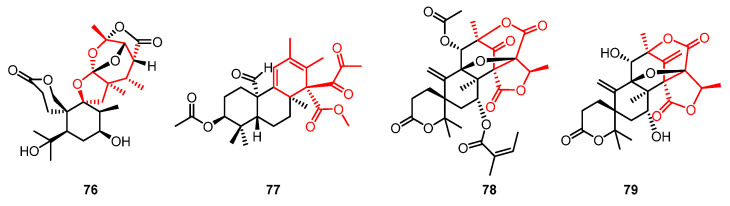
Structures of compounds **76**–**79**.

Simpterpenoid A (**77**) (Figure 21), an unconventional meroterpenoid containing a highly functionalized cyclohexadiene moiety with *gem-*propane-1,2-dione and methylformate groups, was isolated from the fungal strain *Penicillium simplicissimum* MA-332, obtained from the rhizospheric soil of the mangrove plant *Bruguiera sexangular* var. *rhynchopetala* [85]. The intricate polycyclic skeleton is unique in natural sources. Compound **77** exhibited potent inhibitory activity against influenza neuraminidase with an IC_50_ value of 8.1 nM.

**Scheme 12:**
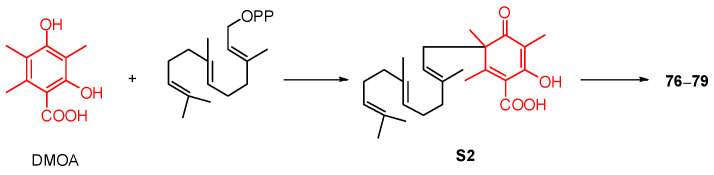
Proposed biosynthetic pathway for compounds **76**–**79** [83].

Two new meroterpenoids, penicianstinoids A and B (**78** and **79**, Figure 21), were obtained from the mangrove-derived fungus *Penicillium* sp. TGM112 isolated from the mangrove *Bruguiera sexangula* var. *rhynchopetala* [86]. Compared with **79**, compound **78** represents an austinoid-like meroterpenoid that is reported for the second time [87], in which a carbon−carbon double bond at C-1′−C-2′ was oxidized to a carbonyl group at C-1′−C-2′. Compounds **78** and **79** showed growth inhibition activity against newly hatched larvae of *Helicoverpa armigera* (Hubner) with IC_50_ values of 200 *μ*g/mL. In addition, **78** and **79** displayed insecticidal activity against *Caenorhabditis elegans* with EC_50_ values of 9.4 (±1.0) and 9.9 (±0.0) *μ*g/mL, respectively. Biogenetically, compounds **76**–**79** are derived from the same intermediate **S2**, which is produced by the combination of a polyketide intermediate 3,5-dimethylorsellinicacid (DMOA) and the terpenoid precursor farnesyl pyrophosphate (FPP), following by a series of further modifications to generate a profile of meroterpenoids with diverse skeletons bearing polycyclic cores. DMOA-based meroterpenoids exhibit diverse structures due to the cyclization of the terpenoid moiety, divergence of post-cyclization modification reactions, and various tailoring reactions (Scheme 12) [83].

**Figure 22:**
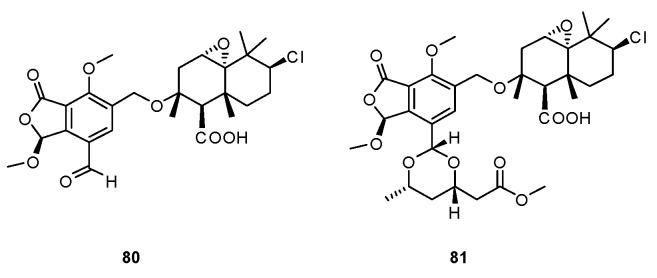
Structures of compounds **80** and **81**.

**Scheme 13:**
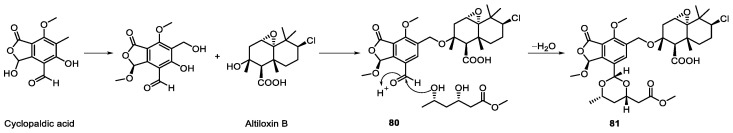
Proposed biosynthetic pathway for compounds **80** and **81** [88].

Two hybrid sesquiterpene-cyclopaldic acid metabolites with an unusual carbon scaffold, namely pestalotiopens A and B (**80** and **81**) (Figure 22), were obtained from the endophytic fungus *Pestalotiopsis* sp. (from the leaves of the Chinese mangrove *Rhizophora mucronate*), together with the known phytotoxin altiloxin B [88]. A plausible biosynthetic pathway of **80** and **81** is proposed (Scheme 13). The cyclopaldic acid and altiloxin B were deduced as precursors.

Indole terpenoids are structurally diverse meroterpenoids containing an indole ring from tryptophan and cyclic sesquiterpenes or diterpene backbone moiety [83].

**Figure 23:**
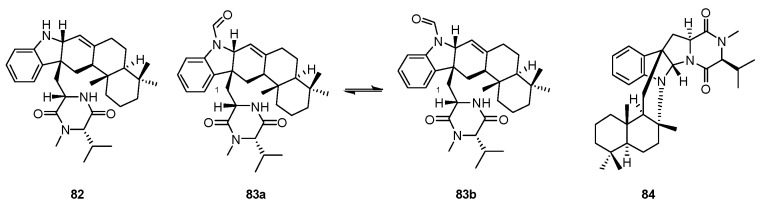
Structures of compounds **82**–**84**.

**Scheme 14:**
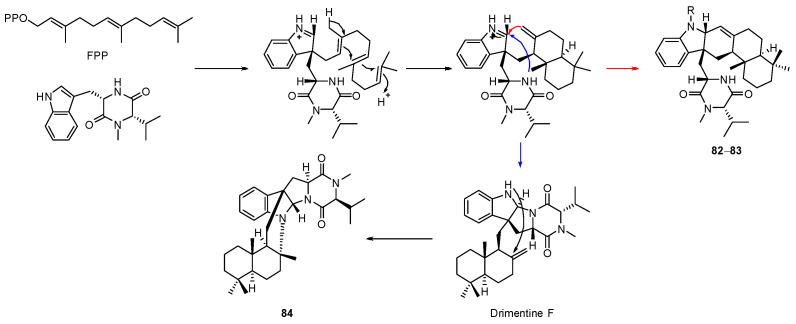
Proposed biosynthetic pathway for compounds **82**–**84** [89].

Three indole sesquiterpenes, indotertine A (**82**) [89] and indotertine B (**83a**/**83b**) [90] (Figure 23) were discovered from actinomycete *Streptomyces* sp. CHQ-64 (derived from the rhizosphere soil of reeds). They possess an unusual skeleton with a condensed ring system made up of a tryptophan-derived indole moiety and a sesquiterpene unit, which represents a new subgroup of indole terpenoids combining amino acid and mevalonate pathways. Indotertine B (**83a**/**83b**) exists as a pair of rotamers about the N−C(O) bond with a 2:1 ratio, inseparable by HPLC because of the dynamic interconversion. The analysis of the NOESY spectrum implied that the formyl−N-1 amide bond was *S-trans* in **83a** and *S-cis* in **83b**. Compound **83** displays cytotoxic activities against HCT-8 and A549 tumor cell lines with IC_50_ values of 6.96 and 4.88 *μ*M. Further chemical investigation of this fungal strain led to the isolation of drimentine I (**84**) [91], containing a rare heptacyclic skeleton formed via two bridging linkages. The pentacyclic product indotertine A (**82**) was hypothetically synthesized by iminium-olefin cyclization. In contrast, tetracyclic product drimentine F could take place from amidic nitrogen by nucleophilic addition to the *α*-position of the indole moiety (Scheme 14). However, cyclization of **84** happened on indol-NH to afford the linkage between C-14 and N-6 of drimentine F. Compound **84** was found to have weak activity against human cervical carcinoma cell line HeLa, with IC_50_ values of 16.73 *μ*M.

**Figure 24:**
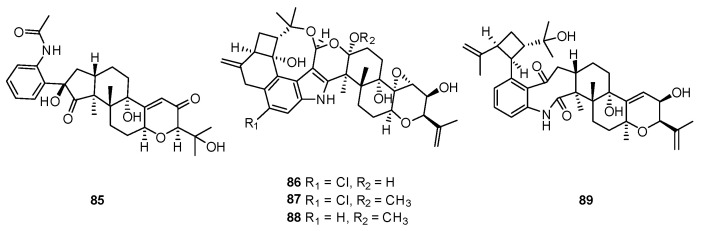
Structures of compounds **85**–**89**.

**Scheme 15:**
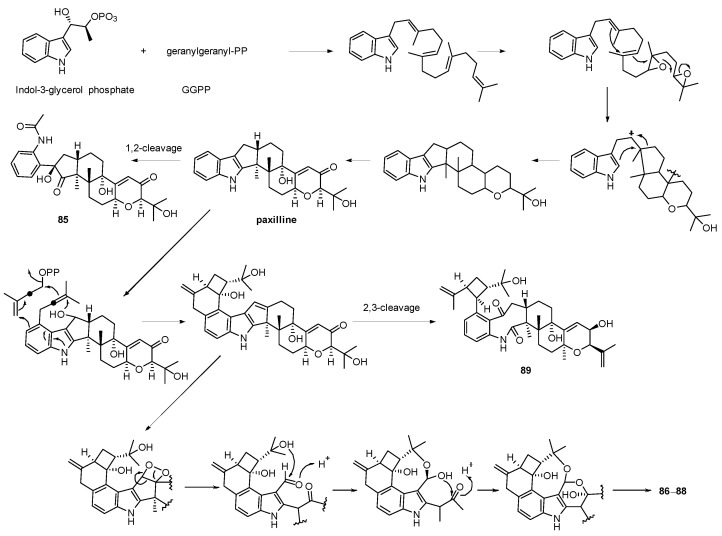
Proposed biosynthetic pathway for compounds **86**–**89** [93].

Secopaxilline A (**85**) [92] (Figure 24) is the first example of indole diterpenoid derivatives possessing a carbon-nitrogen bond cleavage skeleton, which was isolated from metabolites of the aciduric fungus *Penicillium camemberti* OUCMDZ-1492 (separated from the soil and mud around the roots of *Rhizophora apiculata*). A plausible biosynthetic pathway for secopaxilline A (**85**) from paxilline was postulated, (Scheme 15), and the process has been conducted by chemical reactions with a 45% overall yield. Paxilline was derived from the common indole-diterpenoid precursor 3-geranylgeranylindole (GGI) derived from geranylgeranyl pyrophosphate (GGPP) and indole-3-glycerol phosphate [93] (Scheme 15).

The fungus *Mucor irregularis*, isolated from the fresh inner tissue of the mangrove *Rhizophora stylosa*, yields three unusual indole-diterpenes, rhizovarin A–C (**86**–**88**, Figure 24) [94], which represent the most complex members of the reported indole-diterpenes. Even though the main structural elements resemble those of other reported indole diterpenes, the presence of an unusual acetal linked to a hemiketal (**86**) or a ketal (**87** and **88**) unit in an unprecedented 4,6,6,8,5,6,6,6,6-fused indole-diterpene ring system makes them chemically unique. Their structures and absolute configurations were elucidated by spectroscopic analysis, modified Mosher’s method, and chemical calculations. For rhizovarin A (**86**), the biosynthetic pathway may involve more oxidative steps than penitrem A, a known indole-diterpene derived from a paxilline and two isopentenyl-diphosphate units. (Scheme 15) The biosynthetic pathway has been elucidated by reconstitution of the biosynthetic genes in *Aspergillus oryzae* [95]. Another unusual indole-diterpene, containing a complex 6,8,6,6,6-fused ring system, rhizovarin D (**89**), was also obtained in this study. NOESY experiments determined the relative configuration for the stereogenic centers of **89**. Each isolated compound was evaluated for antitumor activity against HL-60 and A-549 cell lines. Compounds **86** and **87** showed activities against the human A-549 and HL-60 cancer cell lines (IC_50_ < 10 *μ*M).

**Figure 25:**
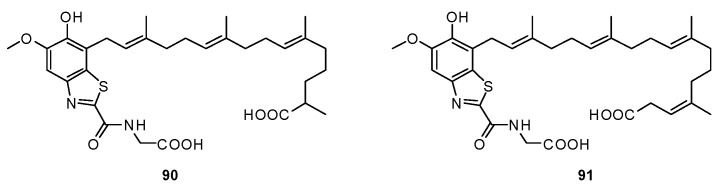
Structures of compounds **90** and **91**.

**Scheme 16:**
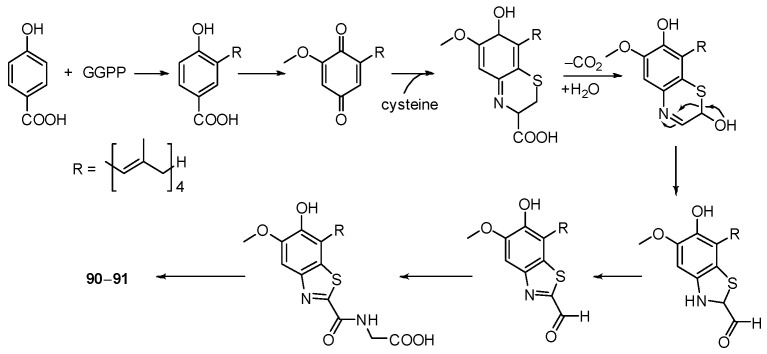
Proposed biosynthetic pathway for compounds **90** and **91** [96].

Bioassay-guided fractionation of the bacterial strain *Erythrobacter* sp. SNB-035 (from mangrove sediments) led to the isolation of erythrazoles A and B (**90** and **91**) [96] (Figure 25). Structurally, **90** and **91** possess an abenzothiazole moiety, which is rare among NPs. Furthermore, **91** arises from four biosynthetic pathways: NRPS, terpene, shikimate, and polyketide. Although combinations of two of the four pathways are common among NPs, four biosynthetic pathways simultaneously involved are extremely rare (Scheme 16).

### 3.3. Alkaloids and Other Nitrogen-Containing Metabolites

#### 3.3.1. Diketopiperazines

Diketopiperazines (DKPs) are an essential group of structurally diverse cyclic dipeptides with significant biological properties [97].

**Figure 26:**
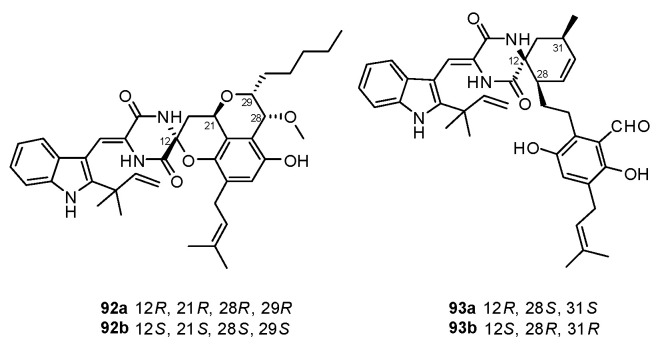
Structures of compounds **92** and **93**.

**Scheme 17:**
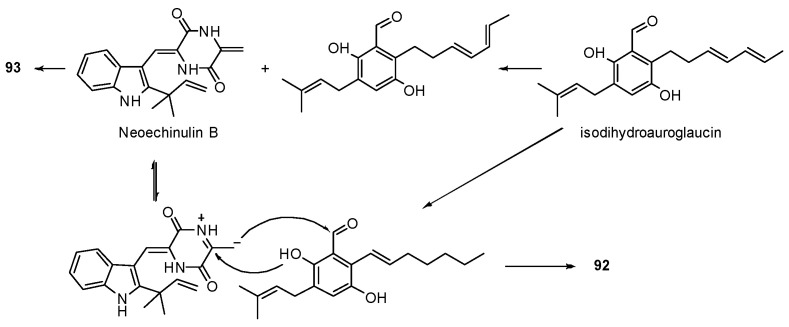
Proposed biosynthetic pathway for compounds **92** and **93** [98].

Effusin A (**92**) (Figure 26) is a spirobicyclic *N*,*O*-acetal derivative with an unprecedented 3′,3a′,5′,6′-tetrahydrospiro-[piperazine-2,2′-pyrano[2,3,4-*de*]chromene] ring system. Besides this, a spiro-polyketide-diketopiperazine hybrid dihydrocryptoechinulin D (**93**) were isolated from a mangrove rhizosphere soil-derived fungus *Aspergillus effuses* H1-1 [98]. Compounds **92** and **93** occurred as racemates. The enantiomers were separated and characterized by online HPLC-ECD analysis, and their absolute configurations were determined by the TDDFT ECD calculation approach. The spirobicyclic *N*,*O*-acetal moiety of **92** could be obtained through a domino ring-closure reaction between the substituted salicylaldehyde moiety in aspergin and the eneamide moiety of the diketopiperazine unit in neoechinulin B [98]. On the contrary, the spirobicycle of **93** is produced by an enzyme-catalyzed regiospecific [4 + 2] Diels Alder reaction (Scheme 17). The cytotoxic effects of **92**–**93** were evaluated, **93** showed potent activity on P388 cells with an IC_50_ value of 1.83 *μ*M. The target of racemic **93** was also evaluated, and the (12*R*,28*S*,31*S*)-**93** enantiomer (**93a**) showed selectivity against topoisomerase I.

**Figure 27:**
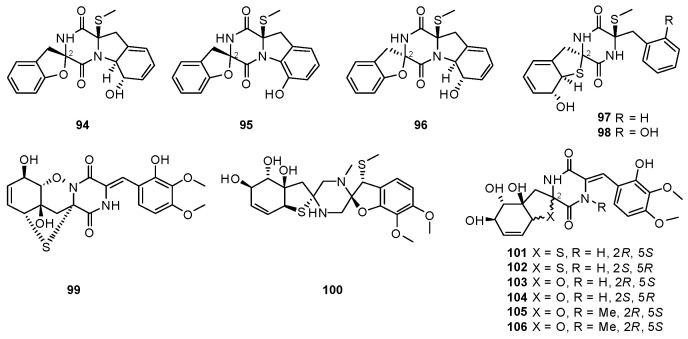
Structures of compounds **94**–**106**.

**Scheme 18:**
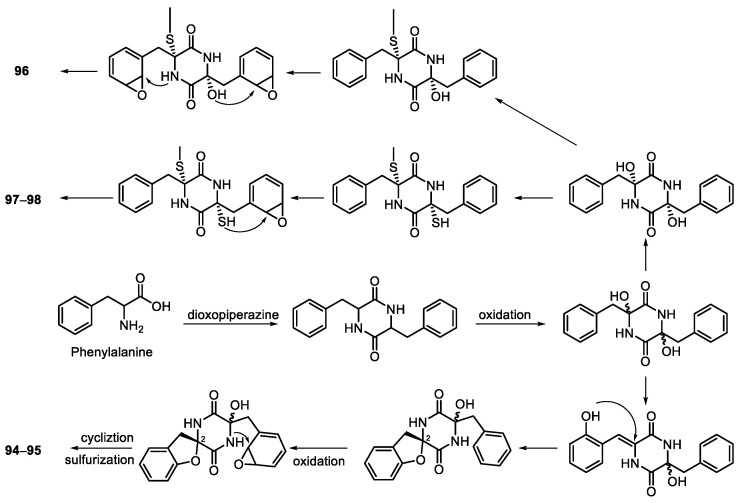
Proposed biosynthetic pathway for compounds **94**–**98** [99,100].

Using the OSMAC (one strain many compounds) approach, a metabolically rich strain of *Penicillium brocae* MA-231 (isolated from mangrove *Avicennia marina*) could produce two new diketopiperazines, spirobrocazines A–B (**94**–**95**) (Figure 27), which had a 6/5/6/5/6 cyclic system with a rare spirocyclic center at C-2 [99]. In addition, a deep-sea sediment-derived fungus *Eutypella* sp. Also yields three new spirocyclic DKPs, eutypellazines N–P (**96**–**98**) [100]. Compound **96** was determined as the C-2′ isomer of spirobrocazine A (**91**). Notably, **97** and **98** are the first compounds isolated from a wild-type fungus to contain a spirocyclic tetrahydrobenzothiophene motif. Furthermore, eight new dioxopiperazines **99**–**106** (penispirozines A-H) were discovered from the mangrove-derived fungus *Penicillium janthinellum* HDN13-309 [101]. The structures of **99**–**104** were similar to eutypellazines O–P (**97**–**88**). They were distinguished by not only the existence of a spiro-thiophane or spiro-furan ring system but also the chirality of the pentacyclic moiety. Moreover, penispirozine A (**99**) had an unusual pyrazino[1,2]oxazadecaline coupled with a thiophane ring system, while penispirozine B (**100**) possessed a 6/5/6/5/6 pentacyclic ring system with two rare spirocyclic centers. Biosynthetically, the precursor to these structurally diverse penispirozines was considered to be the diketopiperazine cyclo-l-Phe-l-Phe (Scheme 18). In addition, compounds **101** and **102** increased the expression of the two relevant phase-II detoxifying enzymes, SOD2 and HO-1, at 10 *μ*M.

**Figure 28:**
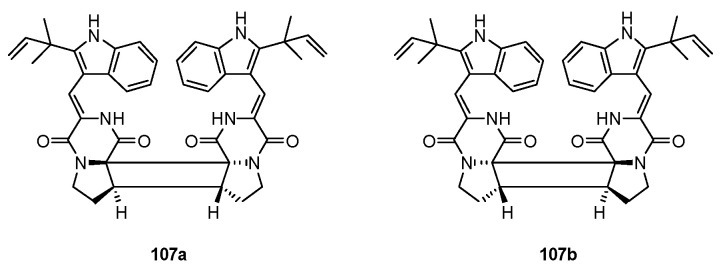
Structures of compounds **107a/b**.

**Scheme 19:**
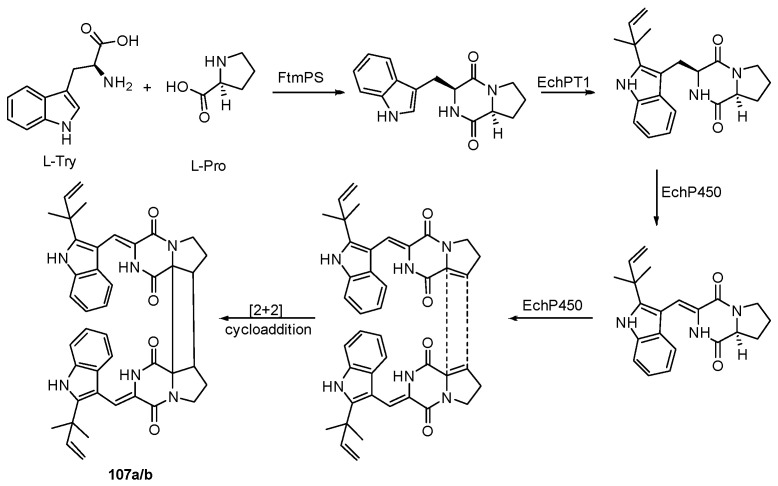
Proposed biosynthetic pathway for compounds **107a/b** [102].

A pair of unusual enantiomeric indole diketopiperazine alkaloid dimers, (±)-asperginulin A (**107a**/**b**) (Figure 28), with an unprecedented 6/5/4/5/6 pentacyclic skeleton, were discovered from the mangrove endophytic fungus *Aspergillus* sp. SK-28, guided by UPLC-HRMS [102]. Chiral-phase HPLC separated the enantiomeric dimers. Their structures, including the absolute configurations, were elucidated by spectroscopic analysis, X-ray diffraction, and quantum chemical calculation. (+)-Asperginulin A (**107b**) exhibited antifouling activity against the barnacle *Balanus reticulatus*. **107** was possibly derived, in vivo, from intermolecular [2 + 2] cycloaddition of its monomer precursor by nonenzymatic processes (Scheme 19).

**Figure 29:**
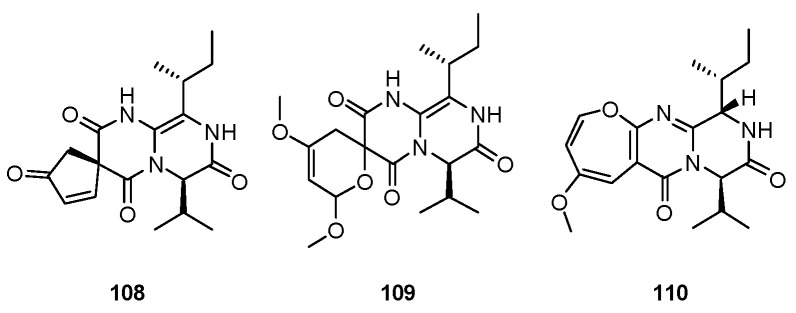
Structures of compounds **108**–**110**.

**Scheme 20:**
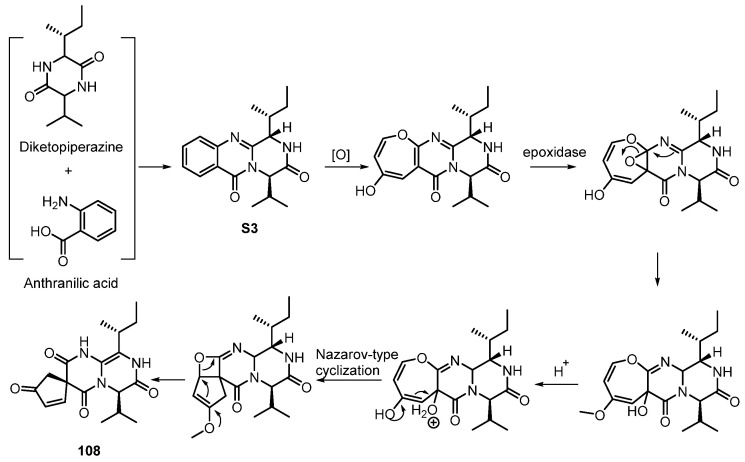
Proposed biosynthetic pathway for compound **108** [105].

A class of pyrazinopyrimidine-type alkaloids, namely pyrasplorines A–C (**108**–**110**) (Figure 29) were discovered from the fungus *Aspergillus versicolor* HDN11-84 [103]. Pyrasplorine A (**105**) represents the first compound with spiro-cyclopentane in pyrazinopyrimidine-type alkaloids. The cyclopentane moiety is common in terpenes but rare in alkaloids and diketopiperazines, and it is only found in maremycins [104]. The structure is probably constructed by the condensation of anthranilic acid with diketopiperazine and followed by successive steps to yield the key intermediate **S3**. Then, compound **108** was derived from the **S3** via a series of reactions [105] (Scheme 20).

#### 3.3.2. Indole and Isoindole Alkaloids Derivatives

Various mangrove fungi produce indole and isoindole alkaloids with a plethora of biologically active. The indole-terpenes which also belong to meroterpenes have been described in Section 3.2.3.

**Figure 30:**
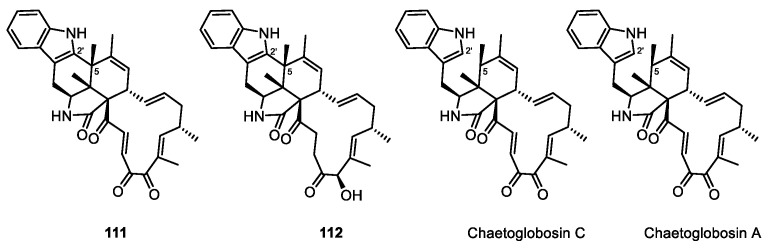
Structures of compounds **111** and **112**, chaetoglobosin A and C.

Cytochalasan alkaloid usually consists of a 10-(indol-3-yl) group, a macrocyclic ring, and a perhydroisoindolone moiety. Chaetoglobosin is one class of cytochalasan alkaloid. The mangrove endophytic fungus *Penicillium chrysogenum* V11 afforded two unusual new Chaetoglobosins, penochalasin I and K (**111** and **112**) [106,107] (Figure 30), with an unprecedented six-cyclic 6/5/6/5/6/13 fused ring system formed by the connection of C-5 and C-2′ of the chaetoglobosin class. Additionally, the biomimetic semi-synthesis of **111** and **112** was successfully carried out from the corresponding co-occurrence analogue chaetoglobosin C and chaetoglobosin A, respectively [107]. Compound **112** displayed significant inhibitory activities against *Colletotrichum gloeosporioides* and *Rhizoctonia solani* (MICs = 6.13 *μ*M, 12.26 *μ*M, respectively), which was better than those of control carbendazim. It also exhibited potent cytotoxicity against MDA-MB-435, SGC-7901, and A549 cells (IC_50_ < 10 *μ*M). In addition, compound **111** exhibited significant cytotoxicity against MDA-MB-435 and SGC-7901 cells (IC_50_ < 10 *μ*M).

**Figure 31:**
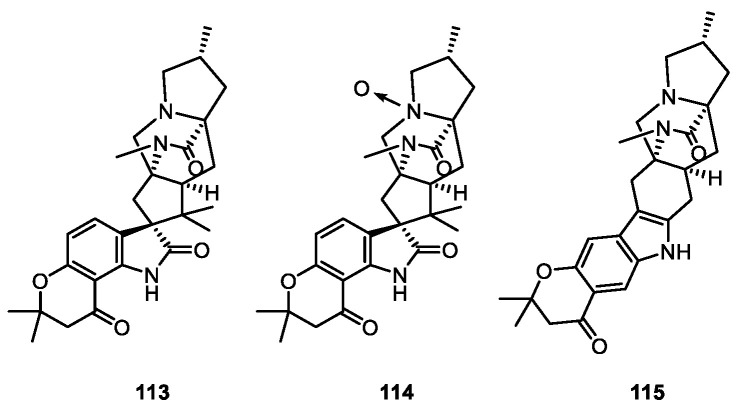
Structures of compounds **113**–**115**.

**Scheme 21:**
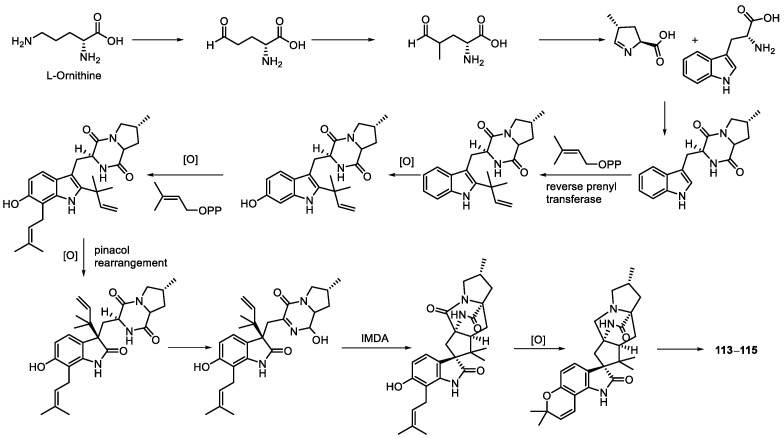
Proposed biosynthetic pathway for compounds **113**–**115** [108].

The typical paraherquamides (PHQs) are prenylated indole alkaloids with diverse ring systems. PHQs are derived from three building blocks: *L*-tryptophan, acyclic amino acid (either proline, *β*-methyl proline, or pipecolic acid), and one or two isoprenyl units. Interestingly, compounds **113**–**115** (Figure 31) (mangrovamides A–C, isolated from the *Penicillium* sp. Separated from a mangrove sediment sample of the South China Sea) feature a bicyclo [2.2.2] diazaoctane core and contain the first documented examples of isoprene derived dimethyl *γ*-pyrone and *γ*-methyl proline, instead of the usual *β*-methyl proline in the PHQ family [108]. A plausible biosynthetic pathway starting from *L*-ornithine to account for the formation of the observed *γ*-methyl proline is outlined (Scheme 21). Moreover, the X-ray data determined the absolute configuration of all chiral centers in **113**. In an activity assay, **115** showed a moderate acetylcholinesterase inhibitory effect with an IC_50_ value of 58.0 *μ*M.

**Figure 32:**
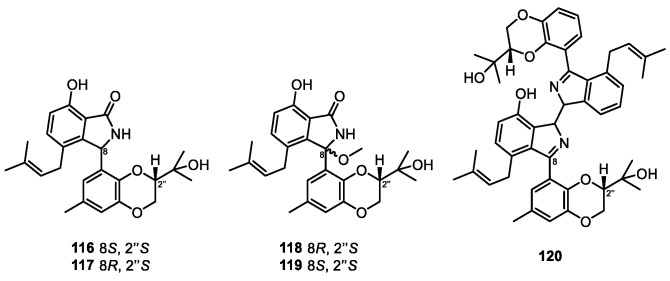
Structures of compounds **116**–**120**.

**Scheme 22:**
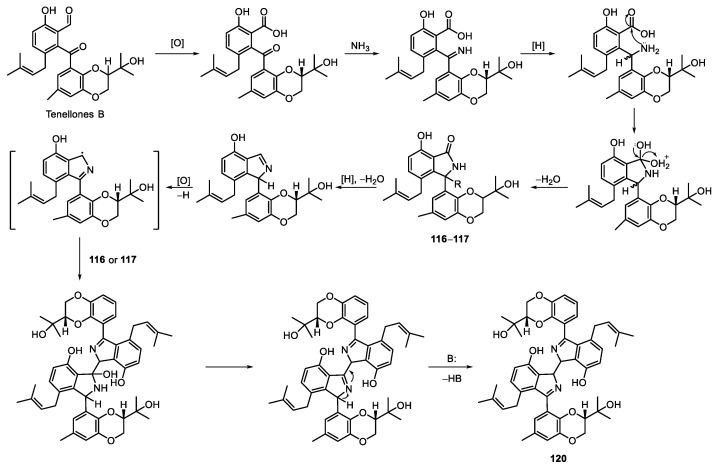
Proposed biosynthetic pathway for compounds **116**–**120** [109].

Diaporisoindoles A and B (**116** and **117**) [109], and D and E (**118** and **119**) [52] (Figure 32), isolated from the mangrove endophytic fungus *Diaporthe* sp. SYSU-HQ3 (from a fresh branch of the mangrove plant *Excoecaria agallocha*) and could be derived from tenellone B, are the first reported examples of isoprenylisoindole alkaloids with a rare 1,4-benzodioxan moiety. In addition, siaporisoindole A (**116**) showed significant inhibitory activity against *Mycobacterium tuberculosis* protein tyrosine phosphatase B with IC_50_ 4.2 *μ*M compared to 22.1 *μ*M for the positive control (oleanolic acid,). Furthermore, **116** and **117** exhibited potent inhibitory activity against NO production in RAW 264.7 cells with IC_50_ values less than 10 *μ*M. Then She et al. continued an extensive study of isolating an unusual diisoprenylisoindole dimer diaporisoindole C (**120**). It was presumed to be derived from compounds **116** or **117** via addition reaction, dehydration, and aromatization (Scheme 22).

**Figure 33:**
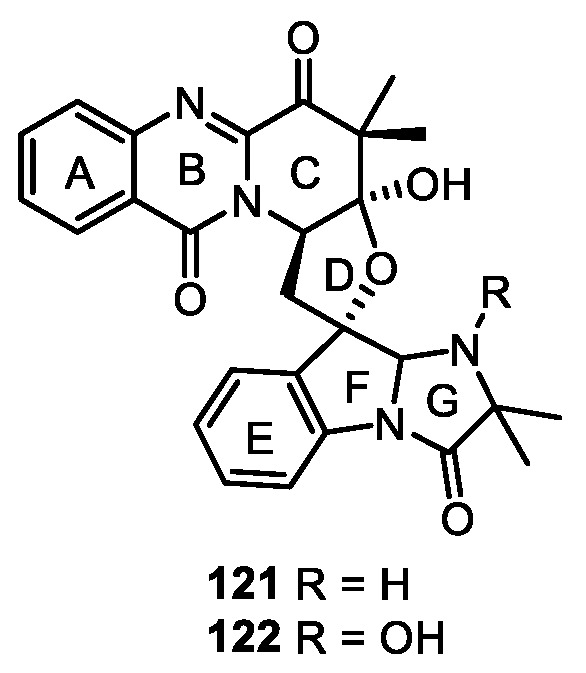
Structures of compounds **121** and **122**.

**Scheme 23:**
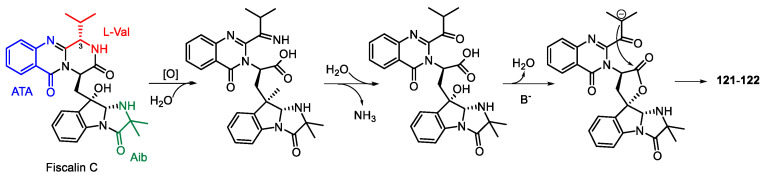
Proposed biosynthetic pathway for compounds **121** and **122 [110]**.

Quinazoline containing indole alkaloids have pyrimidine [2, 1-b] quinazoline and imidazole [1, 2-a] indole groups linked by methylene (and, in some cases, further linked by additional helical Bridges). Two unusual quinazoline-containing indole alkaloids neosartoryadins A and B (**121** and **122**) (Figure 33) along with fiscalin C (a known compound to be related to biosynthesis) were identified from the mangrove endophytic fungus *Neosartorya udagawae* HDN13-313 [110]. Compounds **121** and **122** is a quinazoline-containing indole alkaloid featuring a unique 6/6/6/5 quinazoline ring directly linked to the 6/5/5 imidazolinone ring. **121** and **122** differs from conventional fumiquinazoline alkaloids such as fiscalin C by the unprecedented pyrido[2, 1-b]- quinazoline moiety, which binds to a pyridine (C ring) rather than a pyrimidine ring, in addition to the presence of a unique tetrahydrofuran ring (D ring). It is speculated that **121** and **122** are biosynthesized from *L*-tryptophan, anthranilic acid (ATA), *L*-valine, and 2-aminoisobutyric acid (Aib). The unprecedented C ring was formed by the key intermediate fiscalin C through further modification by oxidation, hydrolysis, water nucleophilic attack, dehydration, deprotonation, and subsequent aldol reaction (Scheme 23).

Streptocarbazoles, the staurosporine analogues with extraordinary cyclic N-glycosidic connections between 1,3-carbon atoms of the glycosyl moiety and two indole nitrogen atoms of the indolocarbazole core, have also been produced by mangrove actinomycetes.

**Figure 34:**
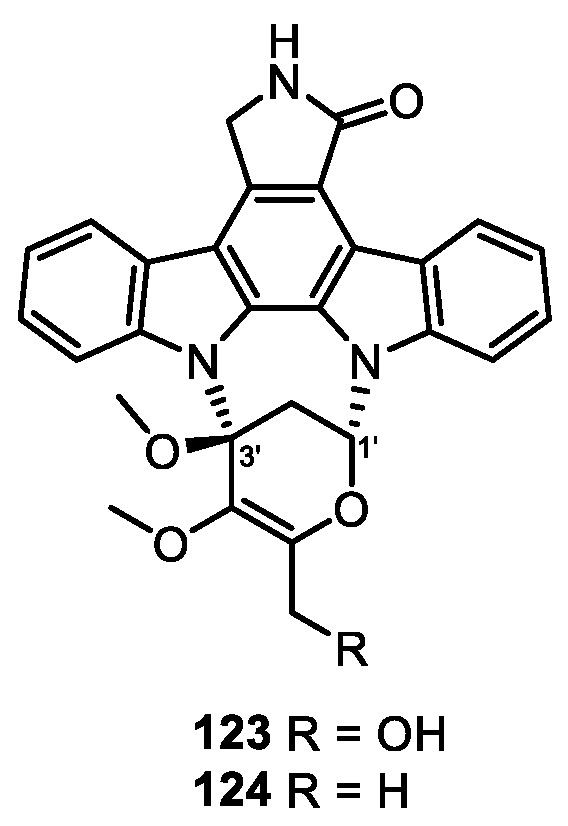
Structures of compounds **123** and **124**.

**Scheme 24:**
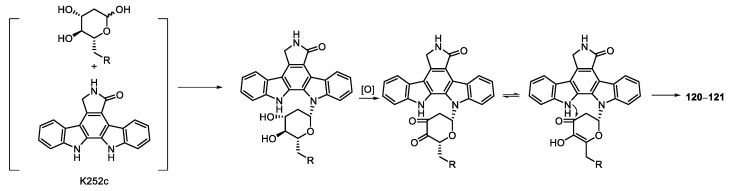
Proposed biosynthetic pathway for compounds **123** and **124** [111].

*Streptomyces* sp. FMA, isolated from mangrove soil collected in Sanya, Hainan Province of China provided streptocarbazoles A (**123**) and B (**124**) [111] (Figure 34). Compound **123** was cytotoxic to HL60, A549, P338, and HeLa cells with IC_50_ values of 1.4, 5.0, 18.9, and 34.5 *μ*M, respectively, while compound **124** was active against P388 and HeLa cells with IC_50_ values of 12.8 and 22.5 *μ*M, respectively. In addition, it was demonstrated that streptocarbazoles A arrest the HeLa cells in the G2/M phase at 10 *μ*M. A plausible biogenetic pathway of **123** and **124** was postulated (Scheme 24). The indolocarbazole unit (K252c) was derived from tryptophan, while the glycosyl moiety was probably developed from 2-deoxy-d-pyranoglucose. Subsequently, the first cloning and characterization of an indolocarbazole gene cluster isolated from *Streptomyces sanyensis* FMA were reported. Indolocarbazole biosynthesis was confirmed by gene inactivation and heterologous expression in *Streptomyces coelicolor* M1152 [112].

#### 3.3.3. Pyridines

**Scheme 25:**
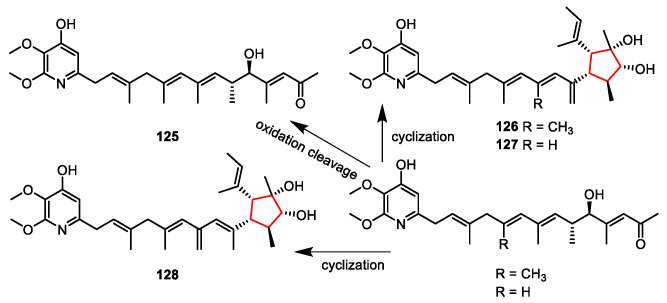
Proposed biosynthetic pathway for compounds **125**–**128** [113].

Piericidins feature a 4-pyridinol core linked with a variable methylated polyene side chain. The strain *Streptomyces iakyrus* SCSIO NS104, isolated from a mangrove sediment sample collected from the Pearl River estuary to the South China Sea, yielded four new piericidin analogues, iakyricidins A–D (**125**–**128**) [113]. Iakyricidins B–D (**126**–**128**) represent a new subgroup of piericidin with C-C cyclization and double bond rearrangements in the polyene side chain. In addition, oxidized side chain piericidin analogue iakyricidin A (**125**) displayed potent antiproliferative activity against human renal carcinoma cell lines ACHN cell with an IC_50_ value of 20 nM. Compound **125** might be derived by oxidative cleavage between C-13 and C-14 of the precursor. In the plausible biosynthetic pathways of **126**–**128**, the most crucial step would be the yet-to-be-identified enzymatic C8-C12 cyclization from the co-occurrence precursor (Scheme 25).

**Figure 35:**
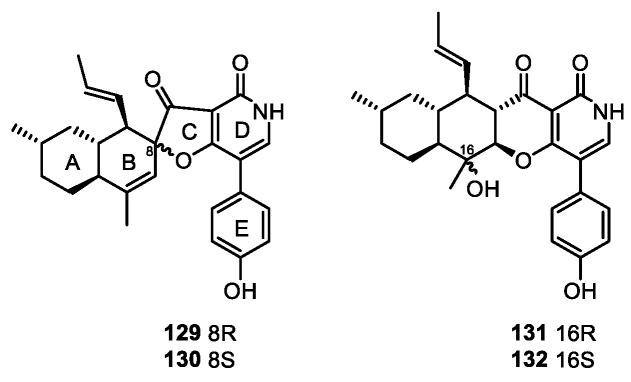
Structures of compounds **129**–**132**.

**Scheme 26:**
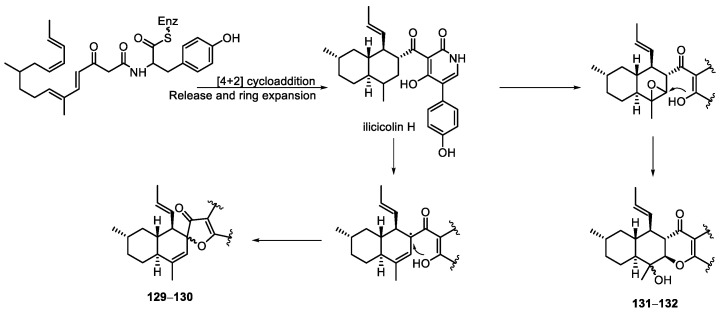
Proposed biosynthetic pathway for compounds **129**–**132** [114].

Chemical investigation of the endophytic fungus *Campylocarpon* sp. HDN13-307, obtained from the root of mangrove plant *Sonneratia caseolaris* led to the isolation of four new 4-hydroxy-2-pyridone alkaloids, namely campyridones A–D (**129**–**132**) [114] (Figure 35), which existed as two pairs of diastereoisomers, featuring an additional C ring between the decalin and pyridone units, represented new ring systems for this family of alkaloids. A plausible biosynthetic pathway for **129**–**132** is postulated with the co-occurrence ilicicolin H as a critical intermediate. Ilicicolin H is a typical 4-hydroxy-2-pyridone alkaloid which was considered to be biosynthesized via tetramic acids formed by hybridizing a polyketide unit to a tyrosine (Scheme 26). Compound **132** exhibited activity against Hela cells with IC_50_ values of 8.8 *μ*M.

#### 3.3.4. Others

**Figure 36:**
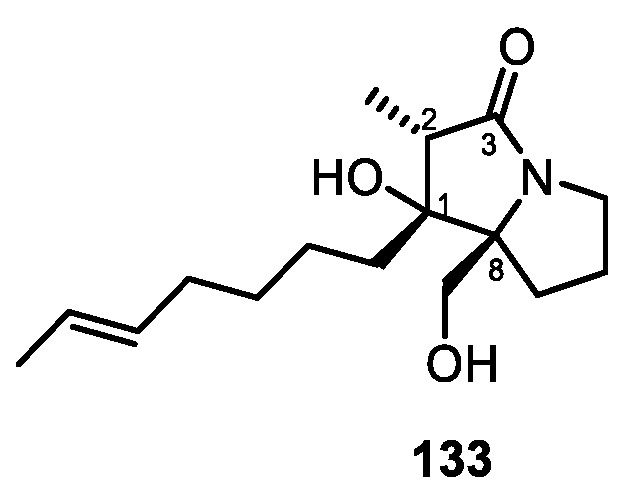
Structure of compound **133**.

**Scheme 27:**
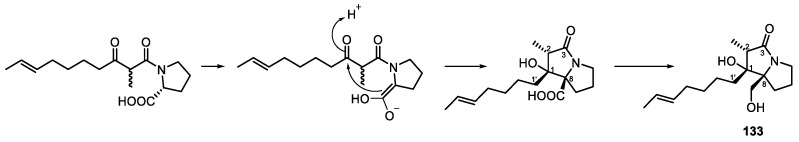
Proposed biosynthetic pathway for compound **133** [115].

A chemical investigation of the fermentation of *Penicillium* sp. GD6, associated with the Chinese mangrove *Bruguiera gymnorrhiza*, resulted in the isolation of an unusual pyrrolizidine alkaloid, penibruguieramine A (**133**) (Figure 36), characterized by an unprecedented 1-alkenyl-2-methyl-8-hydroxymethyl pyrrolizidin-3-one skeleton [115] (Scheme 27).

**Figure 37:**
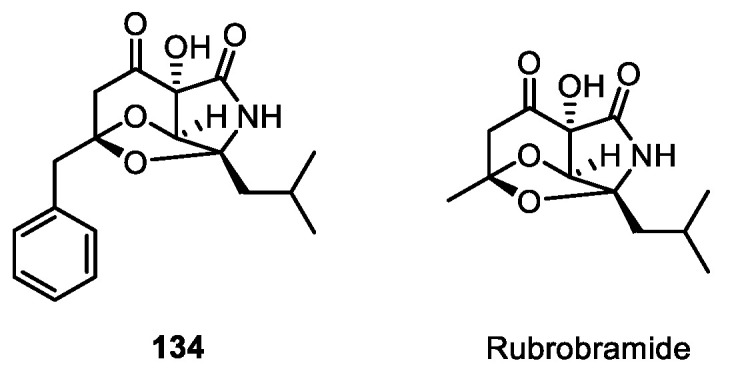
Structures of compound **134** and rubrobramide.

**Scheme 28:**
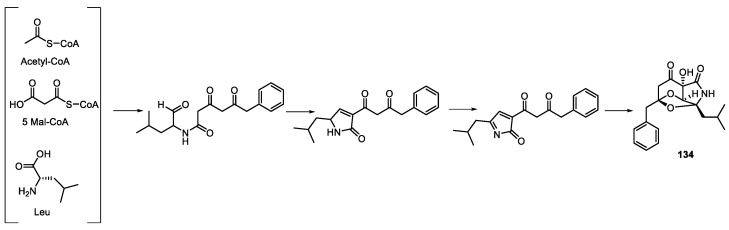
Proposed biosynthetic pathway for compound **134** [116].

Talaramide A (**134**) (Figure 37) is the second example of an alkaloid with a unique oxidized tricyclic system resembling a bird cage, which was obtained from the mangrove endophytic fungus *Talaromyces* sp [116]. The first example was rubrobramide, obtained from the fungus *Cladobotryum ubrobrunnes*cens [117]. **134** was a PKS-NRPS hybrid metabolite derived from acetyl acid, malonic acid, and *L*-leucine. A series of polymerizations, cyclizations, rearrangements, and redox reactions finally afforded the unique oxidized tricyclic skeleton of **134** (Scheme 28).

## 4. Conclusions

In this review, we presented the chemical constituents of the mangrove-associated ecosystem and showcased the diversity of the chemical structures, biological activities, chemical syntheses, and (proposed) biosynthetic pathways.

Structurally diverse secondary metabolites play a crucial role in the discovery campaigns for new NP drug pharmacophores. The mangrove ecosystem is producing various structurally novel compounds that could provide a potent compound library for the identification of lead compounds. Herein, we presented a comprehensive review of 134 mangrove-derived NPs with new carbon skeletons, unique ring systems, or uncommon structural moieties. The majority of them were produced by mangrove-associated microorganisms, and more than 70% were isolated from endophyte fungus, indicating remarkable chemical diversity and interesting bioactivity of the microbial community. The structural novelty and diversity of these metabolites result from the enormous variety of mangrove ecosystems in combination with their potential biosynthetic capabilities. In addition, they display diverse and remarkable biological activities and are frequently reported as antimicrobial and cytotoxic compounds (Table 1 and Table 2), which might attract researchers for further investigations toward chemical synthesis and biosynthesis. Mangrove ecosystems are a rewarding source for producing bioactive substances with novel carbon frameworks and discovering drug lead compounds, attracting pharmaceutical scientists for more in vivo and preclinical studies on these compounds.

In conclusion, through this review, we conveyed that (1) the mangrove-associated ecosystem is still an abundant source of bioactive NPs providing leads for drug development, (2) chemical syntheses of several of the mangrove-associated NPs are completed, but more NPs are to be synthesized and more efficient routes are to be developed, and (3) and the biosynthesis of most of the mangrove-associated NPs remain unclear.

## Figures and Tables

**Table 1:** Structurally unusual secondary metabolites from mangrove flora.

Source	Secondary Metabolites	Reported Activities	Ref
*X. moluccensis*	Thaixylomolin A–C (**1**, **19** and **20**)	Anti-inflammatory	[21]
*X. moluccensis*	Thaixylomolin R (**2**)	NR ^a^	[22]
*X. granatum*	Xylomexicanins E and F (**23** and **3**)	Cytotoxicity	[23]
*X. granatum*	Hainangrantum I and J (**4** and **5**)	NR	[24]
*X. moluccensis*	Trangmolins A–F (**6**–**10**, **17**)	NR	[27]
*X. moluccensis*	Krishnolide J (**11**)	NR	[28]
*X. moluccensis*	Andhraxylocarpins A and C (**12**, **14**)	NR	[29]
*X. granatum*	Andhraxylocarpins A–B and D–E (**12**–**13** and **15**–**16**)	NR	[29]
*X. moluccensis*	Krishnadimer A (**18**)	NR	[30]
*X. granatum*	Xylogranatopyridines A and B (**21** and **22**)	PTP1B inhibitory	[32]
*X. granatum*	Xylomexicanins I and J (**24** and **25**)	NR	[33]
*X. moluccensis*	Xylomolones A–C (**26**–**28**)	NR	[34]
*Excoecaria agallocha*	Agallochaexceorins D–E (**29**–**30**)	NR	[35]
*Ceriops decandra*	Decandrinin (**31**)	NR	[36]

^a^ NR: not reported in references.

**Table 2:** Structurally unusual secondary metabolites from mangrove-associated microorganisms.

Microorganisms	Hosts	Secondary Metabolites	Reported Activities	Ref
*Penicillium commune*	*Kandelia candel*	Peniisocoumarins A and B (**32** and **33**)	NR ^a^	[44]
*Penicillium chermesinum*	Mangrove forest	Penicilliumolide A (**34**)	NR	[45]
*Penicillium* sp.	Mangrove sediment	Penixanthones C and D (**35** and **36**)	NR	[46]
*Penicillium chrysogenum*	*Acanthus ilicifolius*	Penicitols A and B (**37**-**38**)	Cytotoxicity	[48]
*Diaporthe* sp.	*Excoecaria agallocha*	Diaporindenes A–D (**39**-**42**)	Anti-inflammatory	[52]
*Diaporthe* sp.	*Excoecaria agallocha*	Isoprenylisobenzofuran A (**43**)	Anti-inflammatory	[52]
*Penicillium sumatrense*	*Lumnitzera racemose*	Sumalarins A−C (**44**–**46**)	Cytotoxicity	[55]
*Streptomyces* sp.	*Bruguiera gymnorrhiza*	Divergolide A-D (**47**–**50**)	Antimicrobial, cytotoxicity	[56]
*Streptomyces* sp.	Mangrove soil	Hainanmycin A (**51**)	NR	[67]
*Streptomyces* sp.	Mangrove sample	Streptoglycerides A–H (**52**–**59**)	Anti-inflammatory	[69,70]
*Streptomyces* sp.	Mangrove sample	Miharadiened A–D (**60**–**63**)	NR	[71]
*Pseudolagarobasidium acaciicola*	*Bruguiera gymnorrhiza*	Acaciicolin A (**64**), Spiroacaciicolides A–C (**65**–**67**)	NR	[74,75]
*Penicillium bilaiae*	*Lumnitzera racemosa*	Penicibilaenes A–B (**68**–**69**)	Anti-fungal	[77]
*Aspergillus* sp.	Mangrove endophytic	Asperterpenoid A (**70**)	Antituberculosis	[80]
*Aspergillus* sp.	Mangrove endophytic	Asperterpenols A–B (**71**–**72**)	Acetylcholinesterase inhibition	[81]
*Aspergillus terreus*	*Kandelia obovate*	Aspterpenacids A–B (**73**–**74**)	NR	[82]
*Penicillium bilaiae*	*Lumnitzera racemosa*	Chermebilaenes A–B (**75**–**76**)	Antibiotic	[84]
*Penicillium simplicissimum*	*Bruguiera sexangula*	Simpterpenoid A (**77**)	Antiviral	[85]
*Penicillium* sp.	*Bruguiera sexangula*	Penicianstinoids A–B (**78**–**79**)	Insecticidal activity	[86]
*Pestalotiopsis* sp.	*Rhizophora mucronata*	Pestalotiopens A–B (**80**–**81**)	NR	[88]
*Streptomyces* sp.	Mangrove rhizosphere soil	Indotertine A (**82**), indotertine B (**83a/83b**)	Antitumor	[89,90]
*Streptomyces* sp.	Mangrove rhizosphere soil	Drimentine I (**84**)	Antitumor	[91]
*Penicillium camemberti*	Rhizosphere soil of *Rhizophora apiculata*	Secopaxilline A (**85**)	NR	[92]
*Mucor irregularis*	*Rhizophora stylosa*	Rhizovarin A–D (**86**–**89**)	Antitumor	[94]
*Erythrobacter* sp.	Mangrove sediment	Erythrazoles A–B (**90**–**91**)	NR	[96]
*Aspergillus effuses*	Mangrove rhizosphere soil	Effusin A (**92**), dihydrocryptoechinulin D (**93**)	Cytotoxicity	[98]
*Penicillium brocae*	*Avicennia marina*	Spirobrocazines A–B (**94**–**95**)	NR	[99]
*Eutypella* sp.	Deep sea sediment	Eutypellazines N–P (**96**–**98**)	NR	[100]
*Penicillium janthinellum*	Mangrove	Penispirozines A–H (**99**–**106**)	Antioxidant	[101]
*Aspergillus* sp.	Mangrove endophytic	(±)-Asperginulin A (**107a/b**)	Antifouling	[102]
*Aspergillus versicolor*	Rhizosphere soil of *Thespesia populnea*	Pyrasplorines A–C (**108**–**110**)	NR	[103]
*Penicillium chrysogenum*	Mangrove endophytic	Penochalasin I and K (**111**–**112**)	Antibiotic, cytotoxicity	[106,107]
*penicillium* sp.	Mangrove sediment	Mangrovamides A–C (**113**–**115**)	Acetylcholinesterase inhibition	[108]
*Diaporthe* sp.	*Excoecaria agallocha*	Diaporisoindoles A–B (**116**–**117**), diaporisoindoles D–E (**118**–**119**), diaporisoindole C (**120**)	Antituberculosis, anti-inflammatory	[52,109]
*Neosartorya udagawae*	Mangrove endophytic	Neosartoryadins A–B (**121**–**122**)	NR	[110]
*Streptomyces* sp.	Mangrove soil	Streptocarbazoles A–B (**123**–**124**)	Antitumor	[111]
*Streptomyces iakyrus*	Mangrove sediment	Iakyricidins A–D (**125**–**128**)	NR	[113]
*Campylocarpon* sp.	*Sonneratia caseolaris*	Campyridones A–D (**129**–**132**)	Antitumor	[114]
*Penicillium* sp.	*Bruguiera gymnorrhiza*	Penibruguieramine A (**133**)	NR	[115]
*Talaromyces* sp.	Mangrove endophytic	Talaramide A (**134**)	NR	[116]

^a^ NR: not reported in references.
